# Progress in CO_2_ Gas Sensing Technologies: Insights into Metal Oxide Nanostructures and Resistance-Based Methods

**DOI:** 10.3390/mi16040466

**Published:** 2025-04-14

**Authors:** Yash Ughade, Shubham Mehta, Gautam Patel, Roopa Gowda, Nirav Joshi, Rohan Patel

**Affiliations:** 1 Chemistry Department, Parul Institute of Applied Sciences, Parul University, Vadodara 391760, India; 2 Wyant College of Optical Sciences, University of Arizona, Tucson, AZ 85721, USA; 3 Luxembourg Institute of Science and Technology (LIST), 41 Rue du Brill, L-4422 Belvaux, Luxembourg; 4 Research and Development, Amneal Pharmaceuticals, Bridgewater, NJ 08807, USA

**Keywords:** metal oxides, chemiresistive sensing, CO_2_ monitoring, sensing mechanism, sensing performance

## Abstract

The demand for reliable and cost-effective CO_2_ gas sensors is escalating due to their extensive applications in various sectors such as food packaging, indoor air quality assessment, and real-time monitoring of anthropogenic CO_2_ emissions to mitigate global warming. Nanostructured materials exhibit exceptional properties, including small grain size, controlled morphology, and heterojunction effects, rendering them promising candidates for chemiresistive CO_2_ gas sensors. This review article provides an overview of recent advancements in chemiresistive CO_2_ gas sensors based on nanostructured semiconducting materials. Specifically, it discusses single oxide structures, metal-decorated oxide nanostructures, and heterostructures, elucidating the correlations between these nanostructures and their CO_2_ sensing properties. Additionally, it addresses the challenges and future prospects of chemiresistive CO_2_ gas sensors, aiming to provide insights into the ongoing developments in this field.

## 1. Introduction

In contemporary society, the rising threat of global warming and increasing levels of atmospheric pollution are becoming serious issues in day-to-day life [1]. Several gases, including hydrogen sulfide (H_2_S), nitrogen dioxide (NO_2_), and carbon monoxide (CO), significantly contribute to pollution and pose significant risks to both human beings and the environment [2,3,4]. Among these gases, carbon dioxide (CO_2_) is a major contributor to the increasing level of global warming, accounting for almost 76% of greenhouse gas emissions. Due to this, pollution levels are increasing rapidly [1]. In recent years, the outdoor concentration of CO_2_ in the atmosphere has surged by almost 30%, with an annual rate of increase of about 1.5 ppm [5], due to several anthropogenic activities like deforestation, combustion of fossil fuels, industrial processes, and transportation [6]. The global observations of June 2019 show the highest departure from average global land and ocean temperatures since 1880, exceeding temperatures by more than 2 °C in various regions [5]. However, CO_2_ has a dual nature; it is essential for sustaining life through photosynthesis, but in certain cases, it is hazardous for humans as well as the environment; different concentration levels of CO_2_ pose significant health risks such as headaches, fatigue, and respiratory issues. CO_2_ concentrations in outdoor environments of around 400–1000 ppm do not have immediate impacts on health. Furthermore, concentrations above 1000 ppm in indoor environments cause several issues, like drowsiness, discomfort, etc. CO_2_ concentrations up to 5000 ppm have more serious impacts on health, such as shortness of breath, cognitive impairment, increased heart rate, etc. Extremely high concentrations, above 5000 ppm, cause unconsciousness and sometimes even death; such levels also contribute to increasing global warming by trapping heat in the environment, which results in rising sea levels and shifts in ecosystems and biodiversity.

The significance of CO_2_ sensors extends beyond mere detection to enabling precise quantification of this greenhouse gas, which is crucial for implementing effective carbon emission reduction strategies and ensuring human safety in enclosed spaces. Among the diverse sensing technologies available, particular attention should be directed toward metal oxide semiconductor (MOS) sensors due to their exceptional ability to detect CO_2_ through changes in electrical conductivity when gas molecules interact with the metal oxide surface, with recent advances in nanoscale architectures and catalytic additives significantly enhancing their performance. Non-dispersive infrared (NDIR) sensors merit focus for their unparalleled selectivity, utilizing CO_2_’s characteristic absorption of infrared radiation at 4.26 μm, with current research directed toward miniaturization and power optimization to expand deployment scenarios. Electrochemical sensors, particularly those incorporating novel solid electrolytes like NASICON, deserve consideration for their potential to create stable room-temperature CO_2_ sensors suitable for continuous monitoring applications. Metal-organic frameworks (MOFs) require examination for their highly engineerable porous structures that can be precisely tailored for CO_2_ selectivity, potentially addressing cross-sensitivity challenges that plague many sensor types. The continued advancement of these sensing mechanisms represents a critical frontier in addressing the growing global concerns surrounding CO_2_ emissions and their impacts on both human health and planetary systems.

Monitoring and controlling CO_2_ emissions is crucial, necessitating the development of effective detection technologies. The imperative research and innovation in the development of CO_2_ sensors have been driven by many researchers to detect a wide range of concentrations with high sensitivity, rapid response times, and reliable performance. Various techniques, such as non-dispersive infrared (NDIR) sensors [7,8], metal oxide semiconductor (MOS) sensors [9], infrared gas analyzers (IRGAs), and photoacoustic sensors, offer distinct advantages depending on the requirements of specific applications. Despite these advantages, these techniques suffer from various challenges, particularly concerning cost-effectiveness, durability, and energy efficiency [10,11,12]. Along with these, chemiresistive sensors are widely used for CO_2_ detection, offering certain advantages [13]. However, conventional chemiresistive CO_2_ sensors also suffer from limitations such as high cost and limited durability. These sensors often do not perform excellently at room temperature because of weak molecular bonding between carbon dioxide and the surface of the metal oxide sensor. The low temperature further restricts the rapid transfer of charge, which results in small changes in electrical resistance and low sensitivity. In addressing these challenges, the development of new techniques is essential for better results [14]. In this regard, nanomaterials show great potential for CO_2_ sensing due to their promising advantages, including cost efficiency, durability, stability, and room-temperature operation. Additionally, nanomaterials offer enhanced sensitivity, response time, stability, and selectivity. Their application in drug delivery, electrochemical double-layer capacitors, and hybrid supercapacitors makes them important candidates for next-generation CO_2_ sensing technologies [15,16,17]. Due to their increased efficiency and affordability, nanoscale materials like Al_2_O_3_, TiO_2_, CNTs, silica, Cu, and clay are widely used in construction and building products [18].

Chemiresistive devices stand out for their growing popularity and keen interest in CO_2_ sensing, especially when combined with nanomaterials. Nanostructured materials have a large surface-to-volume ratio, which provides an increased number of active sites for gas adsorption reactions. They also offer a compact size, a low cost, long-term stability, low power consumption, and applications in lithium-ion batteries [19,20,21]. Additionally, recent studies on nanomaterial-based sensors have demonstrated the ability to modulate highly reactive crystal facets on the surface of nanostructured sensing layers of metal oxide semiconductor nanomaterials, such as zinc oxide (ZnO), tin oxide (SnO_2_), titanium oxide (TiO_2_), and tungsten oxide (WO_3_) [22]. Furthermore, other materials like conducting polymers and carbon-based semiconductors have also been introduced, expanding the properties of sensing layer materials [23].

**Scheme 1:**
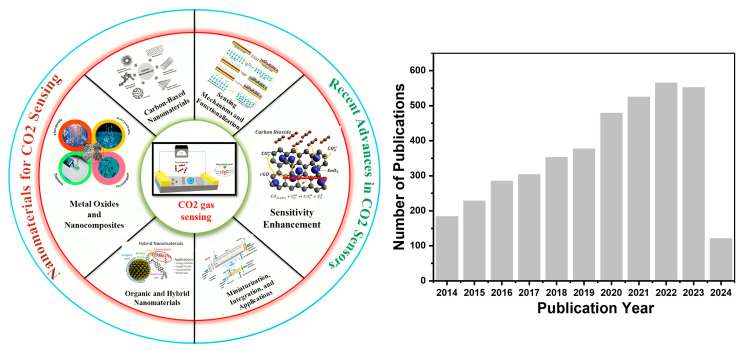
Schematic diagram of CO_2_ gas sensors using functional nanomaterials and the total number of publications in the last decade (Web of Science, accessed in November 2024 using the keyword search (CO_2_ gas sensor and + Nanostructure).

The design and development of sensing materials for CO_2_ detection is the main topic of various review papers on target gases and sensing mechanisms [24]. For example, Molina et al. [25] summarized hybrid and flexible CO_2_ gas sensors, emphasizing materials such as polymers and carbon materials, and discussed their sensing mechanisms. However, there were no efforts to identify additional approaches likely to achieve higher selectivity and stability at room temperature. Lin et al. [5] provided a thorough review of chemiresistive gas sensors, with a focus on CO_2_ detection using metal oxides. They analyzed the sensing mechanisms of these sensors in depth and discussed factors affecting sensing performance, such as humidity and oxygen concentration in the carrier gas. Similar reviews have been produced by Rezk et al. [20] in the last two decades, focusing on nanomaterials-based sensing of CO_2_, primarily in the free gas state and by Hongfeng Chai et al. [26], with an emphasis on stability issues from metal oxides in gas sensing. All of these reviews focused on specific topics without elaborating on recent advances in advanced nanomaterials for gas sensors; the novelty of the present review is that it focuses on advanced metal oxide nanostructure materials, particularly innovative functionalization strategies and heterostructures. Regarding this purpose, the proposed review aims to provide a comprehensive study on the optimization, preparation, and applications of chemiresistive CO_2_ sensors. Many prior papers, as we discussed above, studied general metal oxides but did not dive deeply into heterostructures; they also did not provide any breakthroughs in miniaturization and real-world applications. This paper significantly advances CO_2_ sensing technology through its comprehensive analysis of metal oxide semiconductor-based chemiresistive sensors, detailing their fundamental operating principles and advantages over alternative detection methods. It uniquely breaks down critical performance parameters (sensitivity, response time, recovery time, selectivity, and stability) with precise mathematical definitions, while identifying three key factors that enhance gas sensing properties. The research systematically evaluates both n-type semiconductors (SnO_2_, TiO_2_, ZnO, In_2_Te_3_, and WO_3_) and p-type semiconductors (CuO and NiO), establishing clear correlations between material properties, morphologies, synthesis techniques, and sensing performance. Particularly valuable is its examination of noble metal-decorated metal oxides and heterojunction structures, showing how gold and palladium decorations can improve gas response by up to 50% and how p–n heterojunctions create space charge layers that enhance detection capabilities. This paper bridges the gap between sensor design and operational theory by explaining ionosorption and oxygen vacancy models at the molecular level, providing design guidelines based on the relationship between Debye length and grain size for maximum sensitivity. By addressing CO_2_’s unique detection challenges, comparing various sensor configurations with specific performance metrics, and identifying both knowledge gaps and emerging technologies like flexible sensors and UV-assisted systems, this research creates a comprehensive roadmap for developing next-generation CO_2_ sensors capable of operating effectively at room temperature, a significant advancement for environmental monitoring and industrial applications [27]. The application of functional nanomaterials in CO_2_ sensing, particularly focusing on metal oxide nanostructures, is illustrated in Scheme 1. A literature review using the Web of Science database (up to November 2024) showed an exponential increase in published articles on CO_2_ sensors using metal oxide nanomaterials over the last 10 years. While this review underscores notable progress in nanomaterial-based CO_2_ sensing, it acknowledges the domain’s nature and its constraints in providing exhaustive solutions for all measurement requirements. Instead, this review endeavors to canvas prevailing advancements, delineating strengths, paradigmatic applications, and weaknesses to guide future research efforts in this pivotal realm [28].

## 2. Fundamental Aspects of Metal Oxide-Based Sensors: Key Considerations

Currently, there are various CO_2_ gas sensors available. However, when considering chemiresistive sensors, we find multiple advantages, as discussed above. Chemiresistive sensors provide a simple operation technique that works on the principle of desorption/adsorption of target gases on the surface of metal oxide, affecting electrical changes in resistance [22]. Understanding the basic principles of chemiresistive sensors is crucial for optimizing their performance and expanding their range of applications. This type of sensor measures the changes in electrical resistance of the sensing material when exposed to the target gas. This change in resistance is converted into an electrical signal (i.e., detectable signal), which provides the concentration level of the target gas [29]. With all these advantages, chemiresistive sensors are perfect for detecting CO_2_ gas with good selectivity, low response time, high sensitivity, and a high recovery rate [30]. Therefore, chemiresistive sensors provide more satisfactory results compared to others. Along with the advantages, chemiresistive sensors suffer from some limitations, including susceptibility to interference from other gases, which affects the accuracy of CO_2_ measurements. Another disadvantage is that the sensors may exhibit drift over time, leading to potential inaccuracies in long-term measurements [14]. In addition to susceptibility to interference and potential drift, chemiresistive CO_2_ gas sensors may also face challenges in terms of calibration, sensitivity, and limited specificity to CO_2_.

Semiconductors are composed of conducting polymer, metal oxide, carbon nanotubes, or 2D materials, and when they are exposed to the environment, the gas interacts with the surface of the sensing material, altering its main physical parameters, such as permittivity, conductivity, and work function. Developing low-cost and dependable sensors for room-temperature detection continues to be a major scientific and technological hurdle [31]. For optimal results, gas sensors require certain parameters such as fast response and recovery, high sensitivity, and good selectivity from a sensing material [32,33]. There are some basic parameters that play a key role in achieving high sensing performance, which are as follows:

(1) Sensitivity (S) of a gas sensor: This is defined as the ability of a sensor to detect the presence of target gas when the sensor is exposed to the gas. It is calculated using two methods:

(a) The ratio of resistance in air with respect to gas in air, i.e., S = R_air_/R_gas_. This is the standard way of expressing sensitivity, where R_air_ is the resistance of the sensor in air (absence of target gas), and R_gas_ is the resistance of the sensor in the presence of the target gas. If the value of S is high, then it shows that the material is a good sensor.

(b) S (%) = 100 × (R_air_ − R_gas_)/R_air_. This is another way to calculate sensitivity in the form of percentages. It gives the relative change in resistance exposed to gas and normal air. A positive value of sensitivity implies that the film resistance decreases on exposure to gas and vice versa.

(2) Response time: Response time is defined as the amount of time required for the resistance to attain a fixed percentage (often 90%) of its final value when the sensor is exposed to the gas at full-scale concentration.

(3) Recovery time: Recovery time is the interval during which the sensor’s resistance reduces up to 10% of its saturated value when the sensor is exposed to the gas at full-scale concentration and then placed in clean air. The sensor can be used repeatedly due to its short recovery time.

(4) Selectivity: The most important factor is selectivity because interfering gases can harm the sensor and shorten its lifespan. Thus, the specificity or selectivity of a sensor to an analyte gas is expressed in terms of a dimension that compares the concentration of the corresponding interfering gas and produces the same sensor signal.

$$Selectivity=\frac{Sensitivity\;of\;the\;sensor\;for\;interfering\;gas}{Sensitivity\;to\;the\;desired\;gas}$$

(5) Long-term stability: The capacity of a sensor to display or maintain its performance when used continuously for a long time in a hostile environment. Good sensors are expected to work for several years without exhibiting any drift in the above-mentioned parameters.

**Figure 1:**
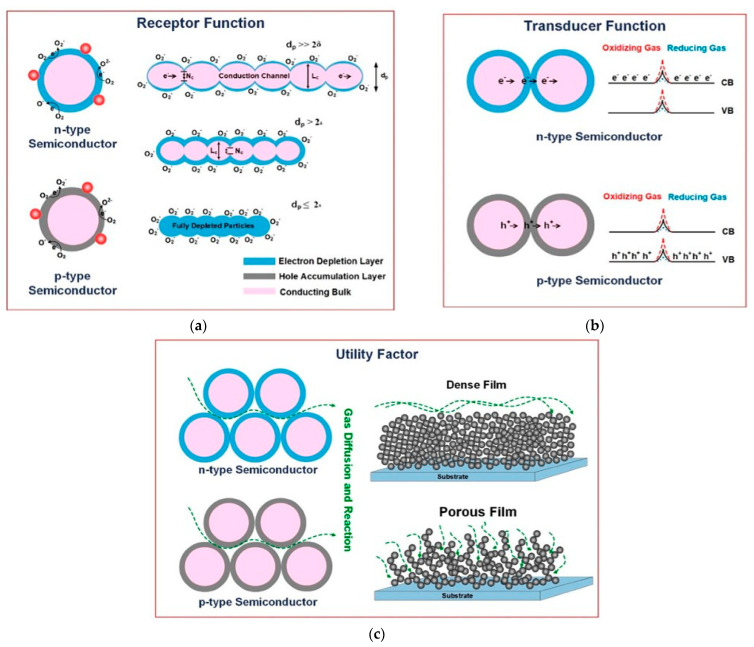
Illustrating three factors of semiconductor gas sensors: (**a**) receptor function, (**b**) transducer function, and (**c**) utility factor [35].

As we know, all these five parameters depend on the sensing material, sensor operating conditions, and the interaction between the gas and the sensor. Therefore, to control these parameters and improve sensing performance regarding sensitivity and selectivity, some other factors or parameters are essential. In addition, there are three factors—namely, the transducer, receptor functions, and utility factor—that enhance the sensing properties of a gas sensor [34]. Gas adsorption, the consequent resistance change, and mechanisms in a metal oxide semiconductor sensor according to these three parameters are illustrated in Figure 1 [35]. The transducer translates the electrical changes arising from interactions between the gases and the sensor material into a signal output in the form of resistance change (Figure 1a). The receptor of a gas detector can recognize oxygen molecules and other gases present in the surrounding environment by the sensing layer. The adsorption of gas molecules is strongly dependent on the surface area of the material (Figure 1b). The utility factor facilitates the passage of gas molecules through the pores in the sensing materials, enhancing the sensor’s response. There are two premier layer configurations that exist in chemiresistive gas sensors, such as compact and porous layers, both of them depicting distinguished procedures for molecular exchange, gas identification, etc. The compact layer (dense layer), known for homogeneous material composition, usually perceives a diminished exterior interface and higher constrained molecular exchange, which provide the outcomes as decreased responsiveness but enhanced long-term performance. On the contrary, the porous layer presents a sophisticated spatial configuration with integrated channel systems, prominently improving the molecular interaction zones and allowing complex detection frameworks between designated chemical species and the sensing material (Figure 1c) [34].

Along with these parameters, there are two techniques for recording the response curve of gas sensing measurements, namely, the static and dynamic environment methods [36]. Using these methods, we measure response curves by continuously recording resistance as a function of time before and after contact with a known concentration of target molecules.

(1) Static environment method

In the static method, the sensing material is positioned within a known volume of a chamber with a gas inlet. After the sensor completely stabilizes in air, a known concentration of the target gas is introduced using a syringe. Under standard atmospheric pressure, the volume ratio of the injected gas determines the final gas concentration. The sensor’s resistance values are recorded over time until saturation occurs, and recovery is observed by exposing the sensor to ambient air after removing the chamber. These measured resistance values are used for further calculations of sensor response/recovery times, response, and sensitivity of the sample.

(2) Dynamic environment method

The dynamic method involves measuring resistance in a continuous flow of the target gas with the proper concentration level. To achieve the desired analyte gas concentration, mass flow controllers (MFCs) are employed for both the target gases through mixing with reference gases like N_2_ or Ar. This dynamic technique allows the recording of sensor responses at various concentrations using MFCs, distinguishing it from the static method. The performance of the sensor is significantly influenced by the working temperature, leading to the attachment of the sensor to a heater regulated by a temperature controller circuit in both systems [37].

### Superiority over Other Technologies

CO_2_, in particular, has garnered significant scientific interest in recent years, primarily due to its connection with climate change and indoor/outdoor air quality. Additionally, CO_2_ is also used as an indicator of patients’ clinical states, food freshness, and fire detection [38]. Therefore, various techniques, such as electrochemical sensors, optical fibers, capacitive techniques, and quartz crystal microbalance, are available for detecting these dangerous gases. However, many of these techniques suffer from disadvantages like low selectivity, high price, low sensitivity, sophisticated design, lack of portability, the need for additional equipment, and more [39].

To overcome these drawbacks, a new and highly effective technique is needed for gas detection. Compared to others, chemiresistive CO_2_ gas sensors are highly selective, sensitive, and reliable. They easily distinguish CO_2_ under different conditions of humidity, temperature, and interference from other gases, making them a subject of intensive research. Chemiresistive tin oxide (SnO_2_)-based sensors hold a privileged position in this research. Several SnO_2_ nanostructures have been used in sensing applications due to their significant features. However, long-term stability and deficient selectivity remain major concerns, especially when the sensor operates at room temperature [38]. Nanomaterials show good potential for chemiresistive sensors in gas sensing [40]. (i) The large surface-to-volume ratio of nanostructured materials provides a larger portion of surface atoms than bulk atoms, contributing more active sites for the analyte gas. (ii) Recent studies reveal that crystal facets with high reactivity can be modulated to be exposed on the surface of the nanostructured sensing layer. The sensitivity of the gas sensor can substantially improve when the grain size of the nanomaterial is roughly equivalent to the thickness of the electron depletion layer (EDL), which is approximately twice the Debye length. Additionally, the superiorities of metal oxide semiconductor nanomaterial sensors are as follows:

(1) Cost-effectiveness [24]: Chemiresistive CO_2_ sensors use low-cost materials and processes for manufacturing, making them more affordable compared to other sensors.
(2) Miniaturization [41]: These sensors can be designed in compact and miniaturized forms to be suitable for integration into portable devices and IoT (Internet of Things) applications.
(3) Low power consumption: These sensors are designed to operate with low power consumption, extending the operational life of the sensor.
(4) Fast response time [42]: These sensors exhibit a fast response time for real-time monitoring and control applications. This feature is crucial for applications where rapid changes in CO_2_ levels need to be detected and acted upon quickly.
(5) Selective sensitivity [43]: The sensing materials in the sensor can be designed to show selectivity for the target gas rather than other gases. Due to selective sensitivity, the sensor’s performance increases as the interference from other gases is reduced, providing actual readings.
(6) Operational stability: Chemiresistive sensors show long-term stability and consistent, reliable performance.
(7) Ease of integration [44]: The integration of chemiresistive sensors into various electronic devices is relatively easier than others, facilitating their adoption in various applications, from industrial processes to electronic devices.
(8) Room-temperature operation [22]: Chemiresistive sensors operate very effectively at room temperature, reducing the need for high-temperature conditions.

While these sensors offer simplicity of operation through desorption/adsorption mechanisms, cost-effectiveness, miniaturization potential, and selective sensitivity, they face significant challenges that require focused research attention. Primary among these issues are susceptibility to interference from other gases, which compromises measurement accuracy; sensor drift over extended periods, leading to potential inaccuracies in long-term measurements; calibration complexities; and limited CO_2_ specificity. This work systematically addresses these challenges by exploring the critical parameters that influence sensing performance, including sensitivity, response time, recovery time, selectivity, and long-term stability. Additionally, this research investigates the impact of three crucial factors, transducer function, receptor function, and utility factor, on enhancing gas detection capabilities. This work further examines how different layer configurations (compact versus porous) affect molecular exchange and gas identification processes, with porous layers offering improved molecular interaction zones for complex detection frameworks. Through comprehensive analysis of both static and dynamic environment measurement techniques, this research offers solutions to advance chemiresistive CO_2_ sensing technology beyond its current limitations.

## 3. Carbon Dioxide (CO_2_)

Carbon dioxide (CO_2_) is significant for both humans and the environment. It is an odorless and colorless gas in the Earth’s environment, produced by many processes, such as the combustion of fossil fuels, human respiration, and industrial activities [45]. In limited spaces with insufficient ventilation, like basements or industrial facilities, CO_2_ can accumulate to levels that pose a health hazard [46]. Unlike gases with distinct odors, the detection of CO_2_ without proper monitoring and controlling equipment is a very challenging task. At certain concentrations, carbon dioxide displaces the oxygen present in the air, resulting in a condition known as asphyxia. High concentrations of CO_2_ in environments can cause symptoms such as headaches, difficulty in breathing, and dizziness. In addition, it can also cause suffocation and respiratory issues. Therefore, the detection of CO_2_ is essential for industrial facilities, laboratories, and confined spaces to ensure the safety of individuals. Furthermore, studies taken to minimize the risks associated with certain CO_2_ levels involve implementing proper ventilation systems, monitoring devices, and adhering to safety standards [47]. Managing and understanding CO_2_ concentrations are necessary for both human safety and environmental management. In regard to CO_2_, many research works have been conducted on sensing CO_2_ gas using various metal oxide-based gas sensors [28].

Table 1 delivers a comprehensive comparison of numerous metal oxide (MO) sensors, including SnO_2_, ZnO, CdO, CuO, and others in multiple forms, such as nanowires, films, and nanoparticles. It illustrates the operating temperature (in °C), gas concentration (in ppm), and response (in minutes)/recovery times (in seconds), indicating how promptly the sensor detects gas and returns to its baseline state. The sensor functions across a broad temperature range from room temperature to 600 °C and can detect gas concentrations from 12 to 10,000 ppm. The essential aspects that can affect the performance variations of CO_2_ gas sensors are gas concentration, material doping, operating temperature, environmental conditions, and material structure and form.

**Table 1:** Illustrating summary of CO_2_ gas sensors based on MO gas sensor.

MO Sensor	Concentration (ppm)	T (°C)	Response (min)/ Recovery (s)	Reference
SnO_2_ film	1000	350	1.16/-	[48]
	2000	240	1.24/4	[49]
	4000	240	1.71/59	
	8000	240	5.86/-	
ZnO film	1000	300	1.01/20	[50]
ZnO nanowires	15 lit/min	200	1.04/40	[51]
ZnO film	400	350	2.86/108	[52]
ZnO nanopowder	5000	400	1.11/38	[53]
CdO nanowires	5000	250	1.03/-	[54]
CdO nanoparticles	4000	250	1.02/-	[55]
CuO film	100	RT	1.04/6	[56]
CeO_2_ nanopellets	80	400	1.32/-	[57]
La_2_O_3_ film	350	250	1.92/73	[58]
TiO_2_ film	1500	450	0.45/55	[59]
Ni-SnO_2_ nanoparticles	100	27	0.04/-	[60]
BaTiO_3_ film	10,000	550	1.04/-	[61]
Ca-ZnO nanoparticles	5000	300	2.0/-	[62]
CoAl_2_O_4_ mesoporous	100	400	0.76/45	[63]
SnO_2_	5000	279	3.0/-	[58]
La_2_O_3_ film	350	321	1.75/73	[64]
MoO_3_	1000	200	0.83/20	[45]
ZnO	500	RT	0.24/15.38	[65]
	1000	RT	0.38/23.73	
	1500	RT	0.52/32.98	
	2000	RT	0.68/43.21	
NiO	500	RT	0.30/21.6	[65]
	1000	RT	0.37/25.47	
	1500	RT	0.44/30.84	
	2000	RT	0.53/35.28	
Ni-ZnO	500	RT	0.24/14.67	[65]
	1000	RT	0.33/22.4	
	1500	RT	0.39/28.32	
	2000	RT	0.45/33.25	
50% La-loaded ZnO	5000	400	1.5/38	[53]
Ni-SnO_2_ nanoparticles	100	275	0.067/-	[60]
ZnO:Ca nanopowders	10,000	450	0.17/10	[66]
LaOCl	2000	260	3.40/-	[67]
Nd_2_O_2_CO_3_	1000	350	4.00/-	[68]
La_2_O_2_CO_3_ nanorods	3000	325	7.08/180	[69]
	2500	320	2.25/120	
LaFeO_3_ nanocrystalline	2000	300	2.19/-	[70]
In_2_Te_3_ film	1000	RT	1.12/-	[71]
ZnO nanostructures	100	350	4.00/5	[72]
	12	350	Poor/-	
BaTiO_3_-CuO film	5000	300	0.3/-	[73]
ZnO (unloaded)	8.5 mbar	100	0.036/-	[74]
La-coated SnO_2_ film	2500	400	0.029/-	[75]
TiO_2_	1500	450	0.70/50	[76]
rGO/TiO_2_	1500	450	0.5/25	[76]
LaOCl-SnO_2_ nanofibers	1000	300	3.7/-	[77]
La_2_O_3_-SnO_2_	1000	350	1.6/-	[48]
La_2_O_3_-SnO_2_ film	500	250	1.42/-	[78]
LaOCl-SnO_2_	2000	425	1.38/-	[79]
LaOCl-SnO_2_ nanowires	2000	400	5.6/-	[80]
CuO-BaTiO_3_	100	456	0.42/-	[81]
CuO nanoparticles	400–4000 (r.h-45%)	25	10.0/-	[82]
	400–4000 (r.h-45%)	50	7.5/-	
	400–4000 (r.h-45%)	65	6.7/-	
	400–4000 (r.h-45%)	80	5.83/-	
	400–4000 (r.h-45%)	95	2.5/-	
	400–4000 (r.h-45%)	150	1.83/-	
	400–4000 (r.h-60%)	25	11.6/-	
	400–4000 (r.h-60%)	50	8.33/-	
	400–4000 (r.h-60%)	65	7.17/-	
	400–4000 (r.h-60%)	80	5.33/-	
	400–4000 (r.h-60%)	95	2.6/-	
	400–4000 (r.h-60%)	150	1.84/-	

RT—room temperature. r.h.—relative humidity.

### 3.1. Detection of CO_2_ by MO Gas Sensors: Working Principle

**Figure 2:**
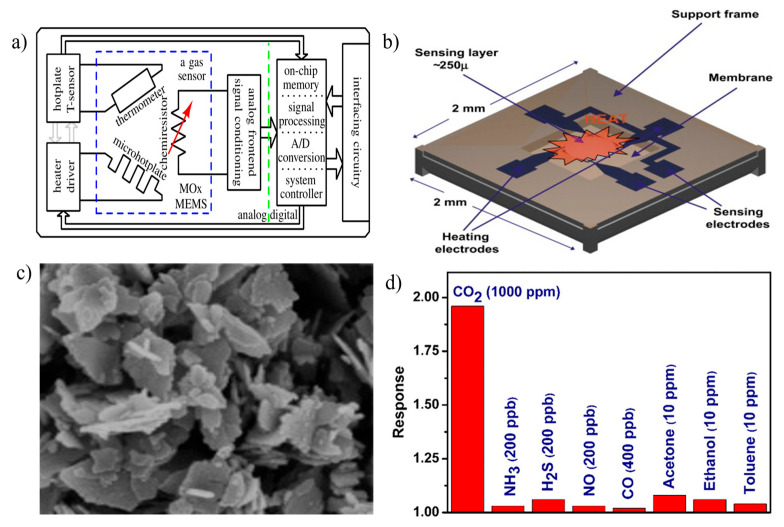
(**a**) General schematical representation of metal oxide gas sensor showing sensor model and interface circuit [30]. (**b**) Illustrating the structure of MOS [85]. (**c**) High-resolution SEM image of ZnO nanoflakes [86]. (**d**) Cross-sensitivity of the sensor for CO_2_ and other gases at 250 °C [16].

The working principle of MO gas sensors is based on changes in electrical conductivity when exposed to the target gas. MOS sensors usually implement semiconductor metal oxide materials like zinc oxide (ZnO) or tin oxide (SnO_2_) [2]. In an MOS sensor, the analog signal is converted to a digital format for the amplification and filtering stages in the sensor’s interface circuit with the help of an analog-to-digital converter (ADC) [83]. Additionally, for handling data processing and communication interfaces such as the Universal Asynchronous Receiver/Transmitter (UART), Inter-Integrated Circuit (I2C), or Serial Peripheral Interface (SPI) for integration with external systems, a microcontroller or digital signal processor (DSP) is included (Figure 2a) [30]. The surface of the metal oxide interacts with the target gas present around the sensor at elevated temperatures of approximately 100–450 °C [22].

Figure 2b depicts the structure of an MOS sensor device, where the metal oxide and electrode layers must be deposited, along with the source and drain regions, contacts, passivation layer, sensing material, and packaging. It contains lead wire, silicon base, electrodes, metal oxide surface, sensor body, etc. As we know, ZnO is also referred to as a good metal oxide for CO_2_ sensing [84]. This is evident from the study by Kanaparthi et al. [16], who demonstrated a chemiresistive CO_2_ sensor based on ZnO nanoflakes synthesized by a simple precipitation process using a zinc precursor and sodium hydroxide at low temperature (Figure 2c). They observed the dynamic response of the sensor to CO_2_ gas at different concentration levels. They also checked the cross-sensitivity of the sensor for other gases. However, they observed that the response of the ZnO-based sensor to CO_2_ was higher than that for other gases at a concentration of 1000 ppm of CO_2_ gas (Figure 2d).

### 3.2. CO_2_ Specific Sensing Mechanism

Metal oxide semiconductor (MOS) sensors detect CO_2_ through a series of physicochemical processes occurring at the gas–solid interface. When the sensor’s metal oxide surface (typically tin oxide, zinc oxide, or tungsten oxide) is heated to an operational temperature of 200–400 °C, oxygen from the air adsorbs onto the surface and extracts electrons from the semiconductor, forming oxygen ions. This creates an electron-depleted region near the surface, establishing a baseline resistance. When CO_2_ molecules interact with this surface, they react with these oxygen species and/or with dopants and surface modifiers specifically added to enhance CO_2_ sensitivity. For CO_2_ detection, these surfaces are often modified with metal carbonates like lithium carbonate, which undergo reversible reactions with CO_2_. As carbon dioxide reacts with these materials, it alters the concentration of charge carriers (electrons) in the semiconductor, causing measurable changes in electrical conductivity [2]. This change in resistance, which is proportional to the concentration of CO_2_ present, is then converted into an electrical signal by the sensor circuitry. The n-type character of the semiconductor makes these sensors particularly responsive to electronic changes at the surface, as even small alterations in electron concentration significantly impact the overall conductivity. As CO_2_ concentration increases, the measured resistance typically increases in properly designed n-type CO_2_ sensors, contrary to the resistance decrease observed when n-type sensors detect reducing gases like CO or H_2_. This distinctive behavior forms the basis for discriminating CO_2_ from other gases in mixed gas environments, though achieving high selectivity remains challenging due to the relatively subtle electronic effects that CO_2_ induces in n-type metal oxide semiconductors [9]. Table 2 highlights the distinctive gas–sensor interaction characteristics across different gas types. CO_2_ stands out with its unique properties, showing minimal sensor engagement compared to reducing and oxidizing gases. While reducing and oxidizing gases demonstrate robust sensing capabilities with high temperature sensitivity and good selectivity, CO_2_ presents challenges in detection due to its chemically inert nature and limited surface interactions. The variations in sensor response, mechanism, and efficiency underscore the complexity of gas detection technologies and the need for specialized sensor designs when dealing with carbon dioxide [87,88].

### 3.3. Pristine Metal Oxide

**Figure 3:**
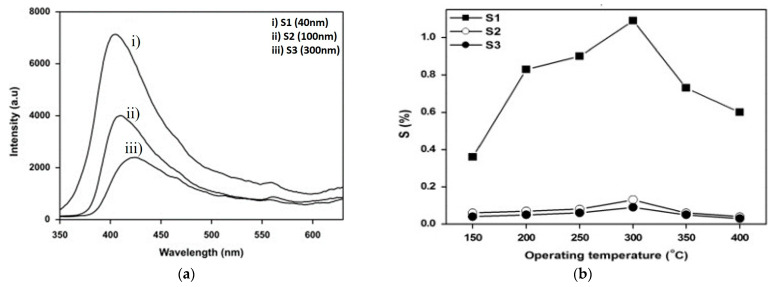

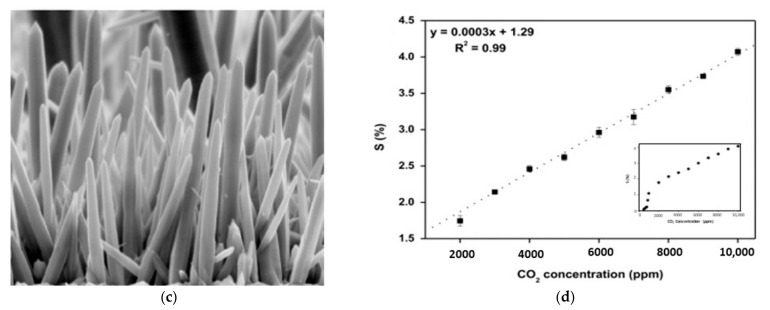
(**a**) Photoluminescence spectra of ZnO film. (**b**) The sensitivity of ZnO sensor for different films for 1000 ppm of CO_2_ concentration at working temperature. (**c**) FE-SEM image of ZnO nanowires [50]. (**d**) Calibrated plot of sensitivity of sensor for different concentrations of CO_2_ [5].

Pristine metal oxide usually refers to unchanged metal oxide material that has not been intentionally altered or enhanced in any way. The commonly used pristine metal oxide sensing components for gas sensors are zinc oxide (ZnO) [84], tin oxide (SnO_2_), silicon dioxide (SiO_2_), titanium oxide (TiO_2_) [59], and zirconium oxide (ZrO_2_). These pristine metal oxides have been used as gas sensors since the 1960s. To enhance their sensitivity, response time, and selectivity in relation to sensing, some efforts have been made by researchers early on. Among various types of metal oxide, ZnO is a widely used metal oxide for CO_2_ gas detection due to its wide band gap of 3.3 eV, high chemical sensitivity, and varied morphologies. Regarding morphologies, Saraswathi et al. [50] synthesized films S1 (40 nm), S2 (100 nm), and S3 (300 nm) of ZnO by the DC sputtering method, while controlling the thickness of the film and grain size, and checked spectra for the intensity of the sample (Figure 3a). It was observed that the sensor’s response was enhanced with the decreasing size of the film (Figure 3b) because of the formation of compressive stress during the annealing of synthesized films. Unfortunately, a low response of 1.01 at 1000 ppm for CO_2_ was observed. To overcome this, improving the response of the sensor is necessary. One versatile way to increase active sites on the surface of the film is through morphological modification. Figure 3c shows the ZnO nanowires that were fabricated. Thereafter, the response obtained from four sets of samples gives RSD values of 1.78% and 0.21% at 1000 and 10,000 ppm of CO_2_, respectively, which indicates good repeatability of the sensor. From this conclusion, it is clear that sensitivity increased rapidly below 2000 ppm (Figure 3d). Similar to ZnO, SnO_2_ is also a widely used metal oxide for CO_2_ gas detection because of its high response, chemical stability, and ease of fabrication. In gas sensors, n-type metal oxide semiconductor (MOS)-based materials are more popularly utilized. Nevertheless, the p-type MOS also shows exceptional sensitivity for specific gases, like carbon dioxide and hydrogen sulfide. There are different material characteristics and gas interaction mechanisms on which the selection of n-type and p-type semiconductors depends, such as stability and durability, compatibility with sensor fabrication, operating temperature, and sensitivity to target gas. Many p-type semiconductors, such as CuO, perovskite oxides, rare earth oxides, etc., have been significantly studied for CO_2_ sensing [91]. Rare earth oxides have gained great attention for CO_2_ sensing due to their extraordinary alkalinity and catalytic properties. LaOCl is a promising material for sensitivity and selectivity in CO_2_ sensors due to the favorable adsorption of CO_2_ on the surface of LaOCl and the formation of carbonate on the lanthanum site. Therefore, n-type and p-type semiconductors can be widely used in environmental monitoring, industrial safety, and breath analysis. Marsal et al. [67] demonstrated the study of the characteristic properties of LaOCl NPs for sensing CO_2_ for a wide range of humidity values. It shows a positive response to CO_2_ at 2000 ppm concentration, with a value of 3.4 under dry air. Therefore, rare earth metal oxycarbonates, like lanthanum dioxide carbonate (La_2_O_2_CO_3_) and neodymium dioxide carbonate (Nd_2_O_2_CO_3_), also exhibit excellent CO_2_ sensing properties due to the formation of the oxycarbonate phase.

In summary, MOS-based sensors are mostly preferred for their straightforward construction, compact size, and affordability. They are also relatively simple to prepare. Despite these advantages, they have some limitations such as a narrow detection range and high power consumption. These disadvantages are attributed to their sensitivity to RH and other interferences, which requires high operating temperatures.

Metal oxides are implemented in chemiresistive sensors that detect changes in electrical resistance. The resistance is proportional to the concentration of the target gas. In chemiresistive sensors, p-type and n-type semiconductors are mostly used. N-type semiconductors have electrons as the predominant charge carriers, while p-type semiconductors have holes as the predominant charge carriers. N-type semiconductors in chemiresistive sensors for CO_2_ detection function via specific electron transfer mechanisms where CO_2_ acts as an oxidizing gas. When CO_2_ molecules adsorb onto the semiconductor surface, they withdraw electrons from the conduction band of the n-type material, which naturally contains excess electrons from donor impurities. This electron withdrawal creates or expands an electron depletion layer at the semiconductor surface, directly increasing the electrical resistance of the material. The magnitude of this resistance increase correlates with the concentration of CO_2_ present, allowing for quantitative detection. Common n-type semiconductor materials used in these sensors include metal oxides such as SnO_2_, ZnO, and TiO_2_, which are often modified with catalysts or dopants to enhance their sensitivity and selectivity to CO_2_. These sensors typically operate at elevated temperatures (200–400 °C) to facilitate the adsorption and electron transfer processes, although recent advances have aimed at developing room-temperature operation capabilities through nanostructuring and surface modifications that increase the active surface area and strengthen the specific interactions with CO_2_ molecules. The overall mechanism represents a direct conversion of chemical information (gas concentration) into an electrical signal.

For instance, n-type metal oxide semiconductors like ZnO, SnO_2_, and TiO_2_ demonstrate superior CO_2_ sensing performance compared to p-type counterparts for several structure-dependent reasons. The n-type semiconductors exhibit advantageous sensing behavior primarily due to their electron-dominated conduction mechanism. When exposed to CO_2_, n-type materials undergo surface reactions where CO_2_ molecules interact with pre-adsorbed oxygen species on the metal oxide surface. This interaction extracts electrons from the conduction band, widening the electron depletion layer and significantly increasing the resistance, a mechanism that directly enhances the sensitivity parameter. In contrast, p-type semiconductors, with their hole-dominated conduction, exhibit smaller resistance changes upon CO_2_ exposure, resulting in lower sensitivity values as calculated by the R_air_/R_gas_ ratio. The nanostructure morphology of these materials further influences the utility factor. ZnO nanorods, for example, offer enhanced surface-to-volume ratios compared to bulk ZnO, providing more active sites for CO_2_ adsorption while maintaining open channels for gas diffusion. This structural advantage directly improves both the receptor function and utility factor, leading to faster response times. Similarly, the crystallographic orientation of exposed facets in n-type ZnO (particularly the oxygen-terminated polar faces) demonstrates higher reactivity to CO_2_ molecules compared to the mixed terminations common in many p-type metal oxides. The stability parameter is also structure-dependent. N-type ZnO and SnO_2_ typically form more stable surface oxygen vacancies that serve as active sites for gas adsorption without significant degradation over time. These vacancies maintain consistent density and distribution during repeated sensing cycles, contributing to the superior long-term stability. Furthermore, the selectivity parameter correlates directly with the band structure of these materials. N-type semiconductors like ZnO possess wider band gaps (3.37 eV for ZnO) and more favorable band alignments for selective interaction with CO_2_ compared to common interferent gases. This structural characteristic enhances the selectivity ratio, particularly when operating at optimized temperatures where CO_2_ adsorption is thermodynamically favored over competing gas species.

Now, oxygen ion formation is normally based or depends on the temperature ($O_{2}^{-}$, $O^{-}$, and $O^{2-}$). The formation of oxygen ions due to changes in temperature is shown below. For example, O_2_ molecules are generated at low temperatures (from RT to 150 °C) as demonstrated in Equation (1).

(1) $$O_{2}+e^{-}↔O_{2}^{-}$$

At higher temperatures, O_2_ molecules dissociate into single or double oxygen ion atoms, removing electrons from the conduction band (CB), as specified by Equations (2) and (3).

(2) $$\frac{1}{2}O_{2}+e^{-}↔O^{-}\;(150–300\;{}^{\circ}C)$$

(3) $$\frac{1}{2}O_{2}+e^{-}↔O^{2-}\;(>300\;{}^{\circ}C)$$

Adsorbed oxygen removing electrons generates a depletion layer (Δ air). The Debye length (L_d_), which shows the depth of the depletion layer, is another crucial concept. The L_d_ in the detector is mainly influenced by the quantity of charge carriers and the working temperature, as shown in Equation (4) [22].

(4) $$L_{d}=\sqrt{\frac{\varepsilon {TK}_{b}}{q^{2}N_{d}}}$$

For example, the Debye length of ZnO nanostructures is generally between 2 and 50 nm and that of SnO_2_ is between 1 and 10 nm. MO sensors typically operate at high temperatures, from 100 to 450 °C, leading to issues like long-term stability degradation due to nanograin aggregation, increased fabrication costs, and high power consumption (100 mW to 1 W). These issues impede their suitability for battery or portable operation. To address this, efforts have been made, such as utilizing low-power LEDs, hybrid materials for low energy consumption due to the reduced temperature, noble-metal functionalization, portable design, low-cost, and energy-efficient sensors for wireless and portable devices [39].

The primary challenges in CO_2_ sensing with metal oxide sensors include the weak interaction between CO_2_ and sensor surfaces, limited selectivity, and high operating temperatures (200–400 °C). CO_2_’s relatively inert nature makes detection difficult compared to reducing gases like H_2_ and CO, which show strong surface catalytic reactions. This weak interaction results in poor selectivity and limited sensing efficiency as highlighted in Table 2. Furthermore, the high temperature requirement leads to issues such as increased power consumption (100 mW to 1 W), long-term stability degradation through nanograin aggregation, and higher fabrication costs, making these sensors impractical for portable or battery-operated applications. Several promising solutions have emerged to address these limitations. Material modifications through doping and composite formation have improved sensitivity and selectivity, with rare earth oxides like LaOCl showing exceptional CO_2_ sensing capabilities due to favorable adsorption properties. Morphological modifications, such as using nanowires and nanoflakes instead of films, have increased active surface sites and improved response times. Recent advances have focused on developing room-temperature operation capabilities through nanostructuring and surface modifications that strengthen specific interactions with CO_2_ molecules. Additional approaches include utilizing low-power LEDs, hybrid materials for reduced operating temperatures, noble-metal functionalization, and designing more energy-efficient sensors suitable for wireless and portable devices. These innovations are critical for expanding the application of CO_2_ sensors in environmental monitoring and industrial safety.

## 4. Types of MOS Sensing Materials

**Figure 4:**
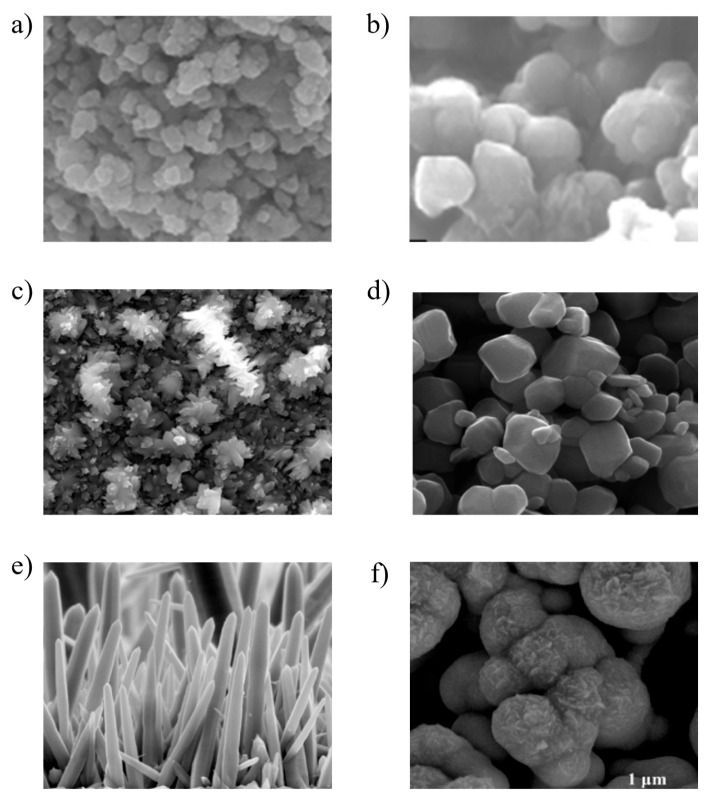
Illustrating the different morphologies for pristine metal oxide. (**a**) SnO_2_ powder [5], (**b**) TiO_2_ NPs [59], (**c**) In_2_Te_3_ thin film [93], (**d**) WO_3_ nanorods [98], (**e**) ZnO NW arrays, and (**f**) ZnO round-shape NP [5].

A survey of various studies on metal oxide semiconductors (MOSs) reveals the advancements and findings in applications of sensing, including n-type materials like tin oxide (SnO_2_) (Figure 4a) [92,93], anatase (TiO_2_) (Figure 4b) [94], In_2_Te_3_ (Figure 4c), tungsten oxide (WO_3_) (Figure 4d), and zinc oxide (ZnO) (Figure 4e,f) [95,96] and p-type materials like CuO [97], Cr_2_O_3_ [95], NiO [96], and Co_3_O_4_ [92], as shown in Table 3. These materials have been extensively studied and are crucial for electronic devices comprising a metal, oxide insulator and semiconductor materials. Here we discuss some metal oxides.

### 4.1. Tin Oxide (SnO_2_)

**Figure 5:**
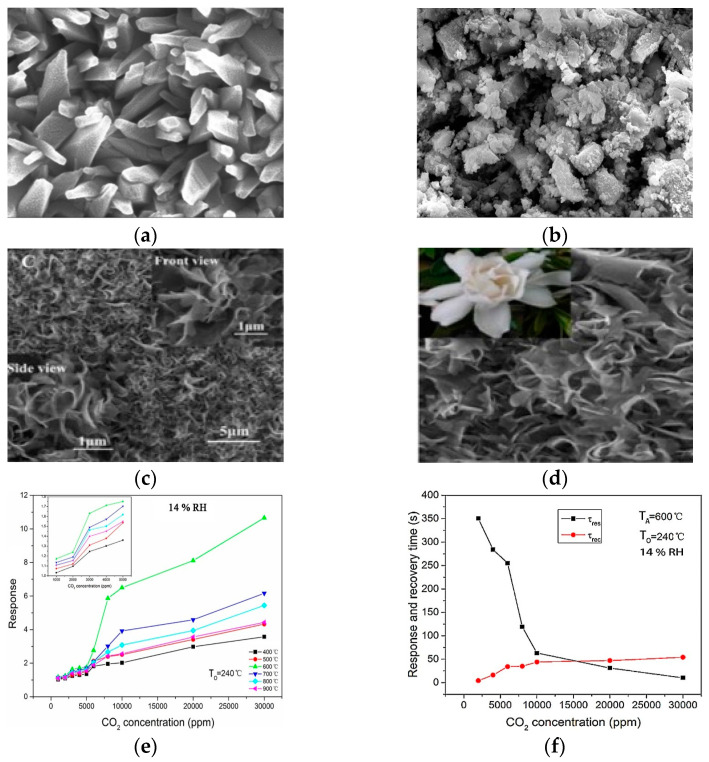

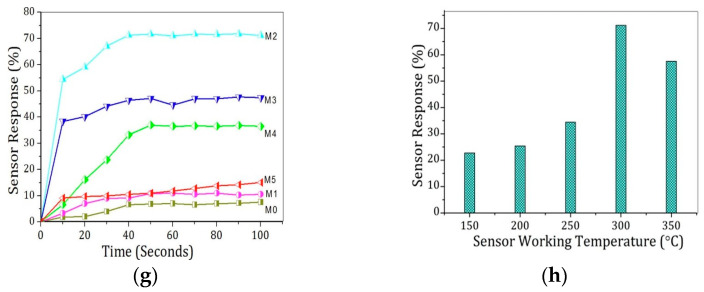
(**a**) FESEM images of SnO_2_ thin film at 1 min irradiated (M2) [100]; (**b**) SEM image of SnO_2_ NPs [101]; (**c**) SnO_2_-grown wonderful nanoflower, showing front and side view; (**d**) comparison between the nanoflower with the gardenia flower [102]; (**e**) at 240 °C, the sensor shows response dependence on the CO_2_ in the presence of 14% RH; (**f**) response and recovery time at 240 °C for SnO_2_ in 14% RH for CO_2_ concentration [49]; (**g**) variation in sensor’s response for MW irradiated and non-irradiated at 300 °C; (**h**) response of sample (M2) at different temperatures [100].

Tin oxide (SnO_2_) is a broadly investigated semiconductor metal oxide for detecting applications and is widely utilized in commercial gas sensors. With a wide band gap of 3.6 eV, it shows both morphological and chemical stability, as illustrated in Figure 5a–d. In n-type semiconductors such as zinc oxide and tin oxide, electrons dominate as charge carriers. Interaction with reducing gases increases conductivity, whereas oxidizing gases exhaust charge-carrying electrons, thus reducing conductivity. The surface states of SnO_2_ act as electron donors or acceptors, impacting electron exchange and shaping a surface near the space charge layer. A porous surface is crucial for enhanced gas interactions, which are essential for effective sensing. Tin oxide-based sensors, owing to their high sensitivity, detect low gas concentrations but suffer from low selectivity. Onkar et al. [99] synthesized a thick film of SnO_2_ using the screen printing technique, and it was measured by a digital micrometer. The synthesized film shows a low response for CO_2_ gas when tested in the presence of reducing gases at a fixed concentration of 600 ppm, with the working temperature varying from 50 to 350 °C. Furthermore, the selectivity of the SnO_2_ thick film sensor is also low for CO_2_ gas at 150 °C for 600 ppm. The thick film shows low sensitivity to CO_2_ gas, but the same work completed by Wang et al. [49] on the sensing properties and mechanism of a nano-SnO_2_ thick-film sensor at different temperatures for CO_2_ showed that the thick film, based on SnO_2_ nanopowders annealed at 600 °C, had a response at a working temperature of 240 °C for different concentrations of CO_2_. The responses at 2000, 4000, and 8000 ppm reached up to 1.24, 1.71, and 5.86, respectively, in the presence of 14% humidity. The response of the sensor for CO_2_ concentration at 14% RH increases with the increasing concentration of CO_2_, as illustrated in Figure 5e. The response and recovery times for the sensor at an operating temperature of 240 °C and annealed at 600 °C are 10 s and 54 s, respectively, for 2000 ppm to 30,000 ppm CO_2_, as shown in Figure 5f. In certain cases, the synthetic technique and change in morphology provide different responses and sensitivity to gas. George et al. [100] prepared a precursor based on SnO_2_ via sol-to-microwave irradiation for various durations. Six samples were synthesized, namely, M0—non-irradiated, M1—0.5 min irradiation, M2—1 min irradiation, M3—1.5 min irradiation, M4—2 min irradiation, and M5—4 min irradiation, giving the sensor response of the samples. M2 shows a higher response than the M0 sample, nearly 70% at 300 °C, because of the maximum ionized formation of CO_2_ at that temperature, as shown in Figure 5g,h. From all the data, it is clearly seen that the pristine metal oxide SnO2-based sensor works at high temperatures for CO_2_ gas. Consequently, this can be overcome by doping or heterojunction to enhance selectivity and sensitivity.

**Figure 6:**
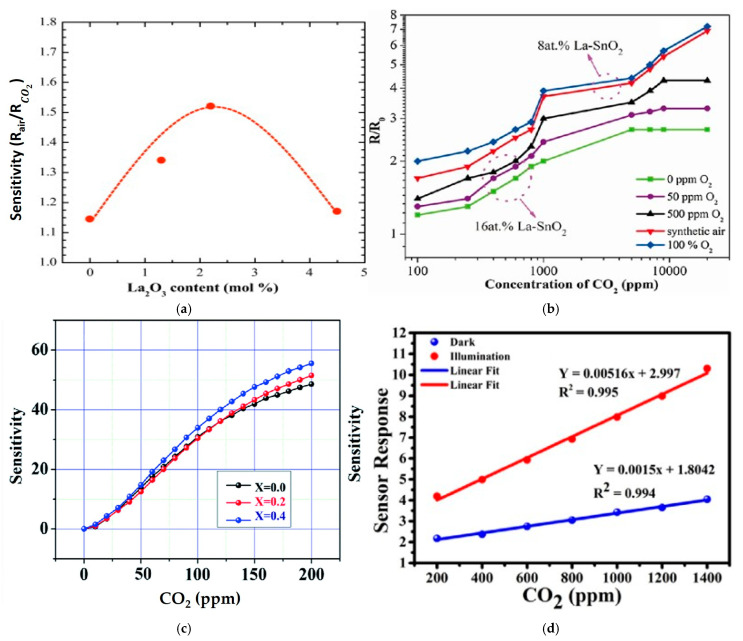
(**a**) SnO_2_ sensors show sensitivity to CO_2_ as a function of La_2_O_3_ concentration; (**b**) 8 at.% LaSnO_2_ and 16 at.% La-SnO_2_ of CO_2_ concentration at 300 °C in different oxygen concentrations [77]; (**c**) The highest response and sensitivity for CO_2_ gas concentrations [60]; (**d**) sensor’s response curve for various concentrations of CO_2_ [104].

Doping of other foreign atoms into the sensor’s structure morphology can improve the performance of sensors. This technique can typically improve the ability of the sensor by changing the material’s electrical and chemical properties. For example, doping can increase the number of charge carriers, modify the surface chemistry of materials, or improve the stability and sensitivity of the sensor. Kim et al. [48] demonstrated the sensitivity of CO_2_ gas by La-doped thick films of SnO_2_ gas sensors, which showed significant results. These results indicate that the sensitivity increased for La_2_O_3_-doped SnO_2_ (1.52) sensors compared to undoped SnO_2_ (1.11). The sensitivity of La_2_O_3_-doped SnO_2_ also increased from 1.14 to 1.52 as the concentration of La_2_O_3_ rose to 2.2 mol%, shown in Figure 6a. Additionally, the La concentration was reported by others, observing a similar bell-shaped behavior of sensitivity. Kim et al. [75] suggested that the improvement in the sensing reaction at the interface between SnO_2_ and La_2_O_3_ is the mechanism for sensitivity improvement. Evaluating the sensor’s selectivity and sensitivity in realistic atmospheric conditions is obtained by analyzing CO_2_ under different concentrations of carrier gases such as nitrogen (N_2_) and oxygen (O_2_). Changing concentrations of N_2_ and O_2_ can affect the behavior of the CO_2_ sensor by enhancing reaction kinetics and adsorption on the surface of the sensor. Achieving accurate CO_2_ detection in various environments and optimizing its performance in different conditions is crucial for understanding. Therefore, many studies have been carried out to investigate the response of CO_2_ sensors under different gaseous environments. Moreover, Xiong et al. [77] studied the detection of CO_2_ under an O_2_ background using LaOCl-doped SnO_2_ nanofibers [103]. Nanofibers of LaOCl-doped SnO_2_ were synthesized by a simple one-step electrospinning technique, and their responses to different oxygen concentrations were analyzed upon exposure to CO_2_ gas. They synthesized films of LaOCl-SnO_2_ with various doping ratios, namely, 4 at. %, 8 at. %, 12 at. %, and 16 at. %, including an undoped sample SnO_2_. They found the best response for 8 at. % La-SnO_2_ with oxygen content in the background with respect to CO_2_. Furthermore, they studied the response of 8% and 16% La-doped SnO_2_ to CO_2_ concentrations between 100 and 20,000 ppm under different oxygen backgrounds at 300 °C, as shown in Figure 6b. Nickel is another dopant with SnO_2_ for improving sensitivity to CO_2_ gas. It improves the selectivity and sensitivity of the sensor and provides accurate detection at lower temperatures. Manikandan et al. [60] utilized a microwave-assisted wet chemical technique to synthesize Ni-doped SnO_2_ nanoparticles. The resulting Ni-SnO_2_ sensor exhibited the highest response and sensitivity to CO_2_ gas concentrations, as shown in Figure 6c. The sensor’s sensitivity ranged between 62 and 73.29, and it attained high sensitivity, having a 4 s response time for 100 ppm CO_2_ gas.

Many investigations regarding undoped and doped SnO_2_ nanostructured materials like Ni or La have been conducted for CO_2_ gas sensors. These elements can heighten the sensor’s selectivity and sensitivity to CO_2_ gas detection by modifying the metal’s surface properties, leading to improved gas adsorption and reaction kinetics. Conversely, to improve sensing performance, facilitating enhanced charge separation and a heightened response to CO_2_ gas, the p–n heterojunction sensors are amalgamated by foreign materials with differing electronic properties. The sufficient charge transfer and surface reactions in the heterojunction architecture contributed to the sensor’s excellent performance. In addition to CuO, CdO also shows a great response and sensitivity for CO_2_ with SnO_2_, as revealed by Singh et al. [104] in 2022. They developed a photo-responsive sensor by nanospheres of SnO_2_ decorated with CdO nanocubes at normal temperature. SnO_2_-CdO heterostructure was prepared via a one-pot hydrothermal method. The sensor showed a great response; the light-induced sensor had a response of 10.29 to CO_2_ at a 1400 ppm concentration at room temperature, with a response time of 18.53 s. This is 2.5 times higher than without illumination, as shown in Figure 6d.

### 4.2. Zinc Oxide (ZnO)

**Figure 7:**
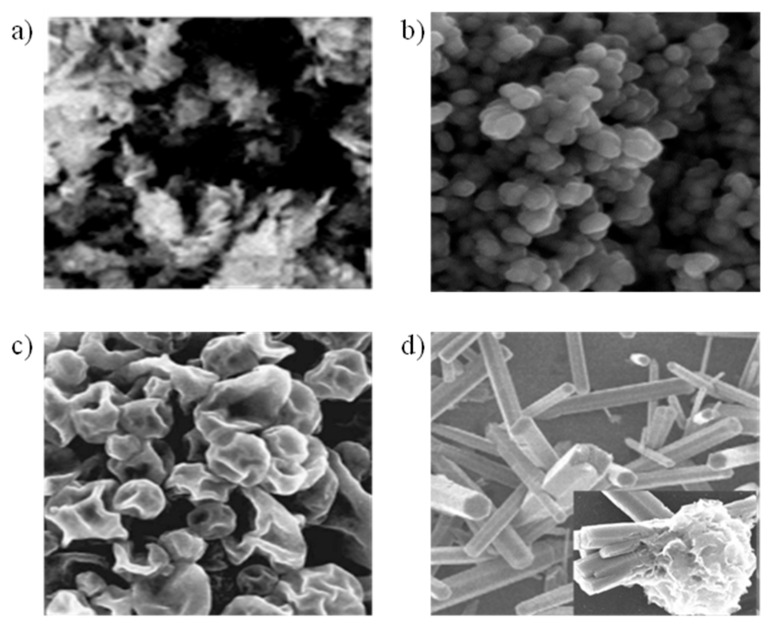
SEM images of different morphologies of ZnO showing (**a**) ZnO nanoflakes, (**b**) ZnO nanocomposites, (**c**) calcined ZnO nanopowder IP-1037 [108], and (**d**) grown ZnO nanorods in acidic condition [109].

**Figure 8:**
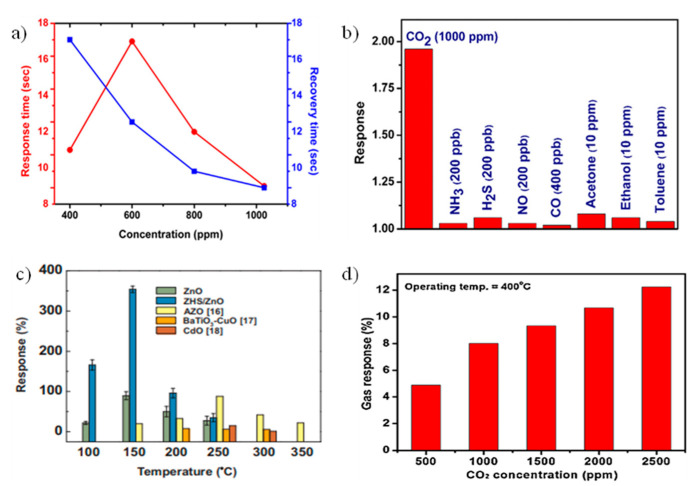
(**a**) Recovery and response curve for concentrations ranging from 400 to 1000 ppm. (**b**) Illustration of cross-sensitivity of sensor for CO_2_ and another gas at 250 °C [16]. (**c**) This image shows a comparison of the responses of ZnO and ZHS/ZnO and other devices at different temperatures [107]. (**d**) Illustration of the response of sensor for different concentrations of CO_2_ (10% La-loaded ZnO) [53].

The advancement in material processing is made possible by nanoscience and accurate molecular manipulation. Nanostructures are especially useful for CO_2_ sensing because their smaller particle sizes provide larger surface areas and the ability to interact with the target gas, making them promising agents. Due to the high exciton binding energy and direct band gap of 3.37 eV, ZnO is more suitable for CO_2_ sensing [105]. ZnO nanocomposites were rediscovered with the development of contemporary nanotechnology and have been synthesized into a variety of morphologies that provide distinct surface areas and exposed crystal facets, including nanoflakes (Figure 7a), nanowires, nanocomposites (Figure 7b), nanopowder (Figure 7c), nanorods (Figure 7d), and nanoparticles. These usually have more active sites for gas adsorption and reactivity in the high surface area morphologies, like nanorods or nanoparticles, which results in increased sensitivity. Different morphologies also affect the electron transport properties of the sensor material and thus alter its recovery durations and responsiveness. Despite the fact that Seiyama obtained the initial results on ZnO’s gas-detecting capabilities in 1962 [106], 1D ZnO nanorods and nanowires, which have better surface chemical properties and improved gas sensing at room temperature and applications in hybrid structures for solar cells, have been the subject of recent research. ZnO nanostructures with surface modification, doping, and light activation offer substantial promise for effective CO_2_ detection. The morphology of various ZnO thin film thicknesses was revealed by Kannan et al. [50] in their thin film investigation. They used DC sputtered ZnO thin films with three distinct thicknesses—40 nm, 100 nm, and 300 nm—to create a chemiresistive CO_2_ gas sensor. They discovered that 300 °C is the ideal temperature for the best response. Over the whole temperature range under evaluation, the 40 nm film exhibited the best response of all the films. Also, at 1000 ppm of CO_2_, the sensor displayed a maximum sensitivity of 1.13% and the same response and recovery time of 20 s. Another class is nanoflakes; however, more research on ZnO nanoflakes reveals improved selectivity and sensitivity for CO_2_ gas sensing. Compared to thin films, nanoflakes have a faster response time. The study by Kanaparthi et al. [16] showed this by employing low-temperature synthesized ZnO nanoflakes to create an ultrafast gas sensor for CO_2_ gas detection. A simple precipitation technique was used to create the nanoflakes at a low temperature. Figure 8a shows that the sensor responded exponentially, with a sensitivity of 0.1135 for 600 ppm CO_2_ and 9–17 s response and recovery time in the 400–1025 ppm CO_2_ concentration range. Additionally, Figure 8b illustrates the cross-sensitivity of the sensor to other gases. They found that the sensor’s response to 1000 ppm is remarkably greater than that of the other gases. This study demonstrates the huge surface-to-volume ratios of ZnO 2D nanoflakes provide exceptional sensitivity and response for CO_2_ sensing. Furthermore, by offering a large surface area for gas contact and promoting electron transport, nanorods can also improve CO_2_ sensing by enhancing the sensitivity and response of CO_2_ sensors. A ZnO nanorods-based sensor with ZHS added as a catalyst was developed by Juang et al. [107]. ZnO nanorods were produced on a silicon substrate in this study, both with and without ZHS microcubes. According to the experimental results, adding ZHS microcubes can increase the high response for CO_2_ by up to 350%. This is higher for CO_2_ sensors that have been reported, either with or without a metal catalyst, as shown in Figure 8c.

Because of numerous qualities, including mass production, low cost, and ease of preparation, ZnO offers a lot of potential for CO_2_ detection in real-world applications. Consequently, there have been numerous attempts to create a pure ZnO-based CO_2_ gas sensor, but the outcomes are not satisfactory. Therefore, doping is an additional method for improving the ZnO-based sensor’s sensing capability. Ca-doped ZnO (CZO) nanoparticles were created by Dhahri et al. [66] for use in high-performance sensors. Nanoparticles of CZO were created using a simple sol-gel process and assessed using TEM and SEM analysis. The sensor exhibits an increase in reaction at 5% CO_2_ as the Ca content rises, with response S = 113 for the highest Ca loading. After examination, the cross-sensitivity was discovered to be higher for CO_2_. Furthermore, ZnO nanopowder loaded with La was introduced as a viable semiconductor material for CO_2_ sensing. La-loaded ZnO nanopowder was created by Jeong et al. [53] using a simple hydrothermal process. They conducted an extensive evaluation of operating temperature, sensitivity, and response/recovery time by changing electrical resistance. According to experimental results, ZnO loaded with 50% La exhibited the highest response to 5000 ppm CO_2_ (65%) at 400 °C. The response of the 10% La-loaded ZnO sensor to varying CO_2_ concentrations is displayed in Figure 8d. The sensor exhibited high sensitivity to CO_2_ gas due to the notable distortion. The collective behavior of La and Zn materials is pivotal for CO_2_ reactivity on the sensor surface and adsorption. Like La-doped sensors, exhibiting strong response and selectivity for CO_2_ detection, the recent study of Kumar et al. [65] demonstrated the impact of Ni-doping on ZnO. They used the sol-gel method to synthesize the NiO, ZnO, and Ni-ZnO compounds. Investigation reveals that the sensor’s response increases when the concentration of CO_2_ increases. The response of Ni-ZnO was examined for all three manufactured sensors for CO_2_ at RT for various concentrations (500, 1000, 1500, and 2000 ppm). Results showed that the sensors’ sensitivity at 500 ppm was 0.0024, 0.0025, and 0.003 for ZnO, NiO, and Ni-ZnO, respectively. This finding suggested that nanomaterial-based sensors exhibited good sensing characteristics. The response times of all the sensors range from 14 s to 41 s, while the recovery times range from 15 s to 44 s. All of these studies show that doping is a more effective approach to improving sensor performance.

ZnO-based heterojunctions become more attractive platforms for sensing and optoelectronic applications due to their unique qualities and adaptable functions. They are combined with other semiconductor materials like CaO, SnO_2_, CuO, and TiO_2_ to prepare a new heterojunction that improves charge carrier dynamics and customized electrical band structures. In order to detect CO_2_ gas, Joshi et al. [110] converted zinc hydroxide carbonate to CaO-ZnO. Nevertheless, CaO-ZnO heterostructures showed a notable sensitivity of 26 at 150 °C for concentrations ranging between 100 ppm and 10,000 ppm. These heterostructures were synthesized for low-temperature CO_2_ sensing. Regarding 500 ppm CO_2_ gas, the CaO-ZnO heterostructures (25CaZMS) showed an encouraging sensitivity of 77% and selectivity of 98% outcomes. Additionally, the sensor’s sensitivity to CO_2_ gas was shown to be higher when selectivity studies including 10 typically occurring gases were undertaken, and their sensing ability was evaluated in both dry and wet situations. A recent study on ZnO/CuO heterojunctions for CO_2_ sensing at RT was developed in another investigation of ZnO-based heterojunction sensors presented by Keerthana et al. [111]. These sensors also demonstrated an excellent response to CO_2_ of 9.7% at 1000 ppm, with recovery and response times of 2.3 min and 1.3 min.

### 4.3. Other Metal Oxide (In_2_Te_3_, CuO, NiO, WO_3_, and TiO_2_)

**Figure 9:**
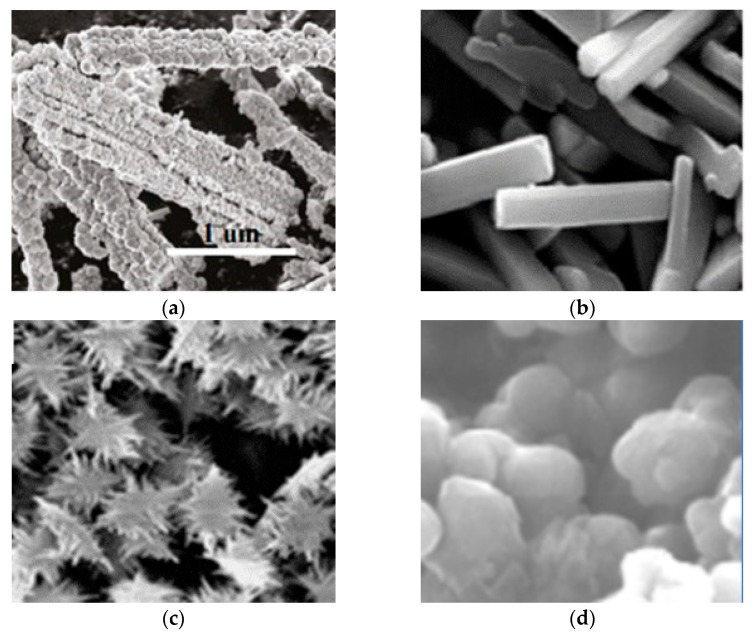
SEM images of metal oxide. (**a**) In_2_Te_3_ hierarchical structure [112]; (**b**) CuO nanorods; (**c**) dendrite-like structure of CuO [113]; (**d**) TiO_2_ nanoparticles [59].

**Figure 10:**
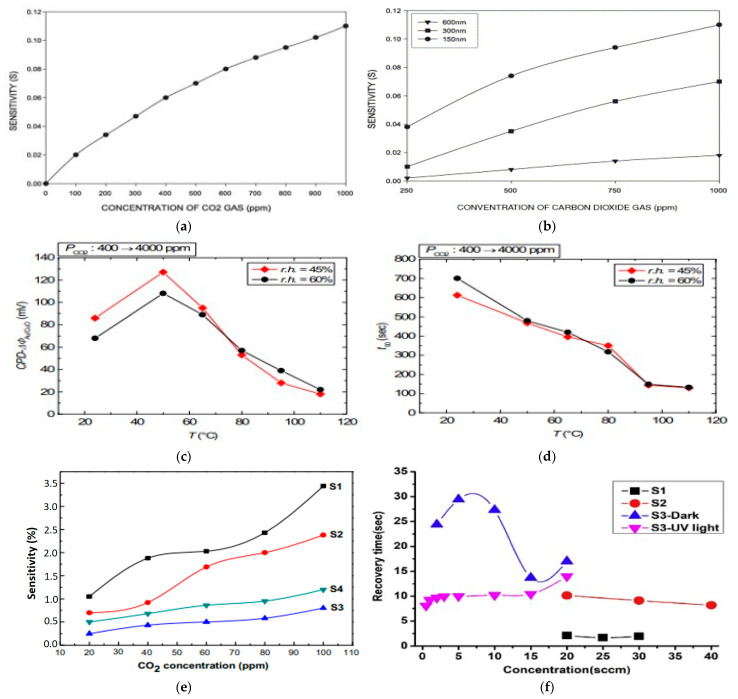
(**a**) Variation in In_2_Te_3_ thin film gas sensor’s sensitivity at various CO_2_ gas concentrations. (**b**) The impact of thickness on the In_2_Te_3_ thin film gas sensor’s sensitivity for various CO_2_ gas concentration values [71]. (**c**) Work function response of CuO NPs for a 400–4000 ppm CO_2_ exposure with two different humidity levels as a function of temperature. (**d**) Temperature dependence of work function response times [82]. (**e**) The sensitivity of the sensor to CO_2_ at room temperature for different samples [56]. (**f**) S1, S2, S3-Dark, and S3-reaction and recovery time under 365 nm UV light [42].

Other metal oxides such as In_2_O_3_ (Figure 9a), CuO (Figure 9b,c), NiO, TiO_2_ (Figure 9d), and WO_3_ are also promising agents for CO_2_ sensing. Their physiochemical properties make them suitable for sensing and monitoring CO_2_ gas with high selectivity and sensitivity. For example, NiO provides high stability and selectivity, while In_2_O_3_ shows notable electron mobility and surface reactivity. CuO is well known for its surface chemistry and high sensitivity to CO_2_, and TiO_2_ and WO_3_ exhibit high response qualities because of their electronic band structures and surface interactions. Additionally, In_2_Te_3_ is a promising agent for CO_2_ gas sensing due to its distinct electrical characteristics; it has a 2.8 eV variable band gap that allows for selective CO_2_ detection, while its large surface area improves gas interaction [112]. The potential of In_2_Te_3_ in creating effective and sensitive CO_2_-detecting devices is still being investigated. Using the flash evaporation approach, Desai et al. [71] produced In_2_Te_3_ thin films of different thicknesses at 473 K substrate temperature on ultrasonically cleaned glass substrates. The resulting In_2_Te_3_ thin film sensors have a detection range between 100 ppm and 5000 ppm and demonstrate remarkable sensitivity to CO_2_ gas (Figure 10a). The sensor was noteworthy for its quick response time and its outstanding stability in a variety of temperature settings. Also, Figure 10b shows the effect of different film thicknesses on sensitivity. In_2_Te_3_ thin film gas sensors also have benefits including low power consumption, good selectivity, and integrated circuit compatibility. Additionally, radiation is a major factor in gas detection, as revealed by Matheswaran et al. [93], who made a substantial contribution to our understanding of the impacts of radiation on the sensing characteristics of indium telluride (In_2_Te_3_) thin films. According to their research, irradiation significantly improved In_2_Te_3_ thin film sensitivity to CO_2_ gas, with notable improvements observed in sensitivity over a range of 100 ppm to 1000 ppm concentrations at different samples (pristine, 1 × 10^12^, 3 × 10^12^, 1 × 10^13^, and 3 × 10^13^). Following the radiation, the sensor’s response time decreased to about 3 s, suggesting a significant improvement in detection effectiveness. Moreover, the sensor demonstrated improved stability in a range of temperature settings, indicating its potential for accurate CO_2_ detection in various environments.

For p-type semiconductor materials, copper oxide (CuO) has garnered significant interest due to its various applications, with one of the most well known being sensing and monitoring. Optimizing inter-particle connections is crucial for CuO to achieve enhanced gas sensitivity. CuO is a notable example of a p-type semiconductor metal oxide that has exceptional selectivity for CO_2_ gas, as do NiO, Cr_2_O_3_, and Mn_3_O_4_. P-type sensors also have the benefit of being resistant to dampness. Numerous CuO morphologies, such as those resembling urchins, fibers, and nanorods, have been effectively synthesized using techniques like hydrothermal and microwave processes. In particular, CuO nanoparticles made by the hydrothermal process exhibit CO_2_ gas sensitivity. According to Tanvir et al. [82], who studied the work function of CO_2_ sensing using CuO nanoparticles in relation to humidity and temperature, CuO NPs have successfully optimized CO_2_ gas sensing behavior when temperature and humidity are combined. This development is important because it will lead to the creation of low-cost CO_2_ gas sensors. According to his research, CuO NPs’ dependence on temperature and humidity in response to 400–4000 ppm concentrations of CO_2_ gas is higher at constant humidity levels of 45%, but the work function decreases as humidity levels rise. Similarly, the temperature response function is high at room temperature and decreases as temperature rises (Figure 10c). The conclusion drawn is that, as the temperature rises, it serves as an opposing factor for humidity dependency and nullifies the variance caused by humidity of function response, as evidenced by the interesting fact that the function shows higher sensitivity of the sensor than that observed for room temperature (Figure 10d). Another study by Abdelmounaim et al. [56] on nanostructured CuO films in relation to CO_2_ gas sensing properties, synthesized by spray pyrolysis, obtained different samples of CuO films (S1 0.05 M, S2 0.1 M, S3 0.2 M, and S4 0.3 M) in relation to CO_2_ concentrations at room temperature. Figure 10e illustrates the sensitivity varied at various CO_2_ concentrations at room temperature. In conclusion, the sensitivity of sample S1 exhibits the highest sensitivity to varying concentrations of CO_2_. Also, porosity gradually disappearing produces S3 and less sensitive S4 films.

Although tungsten oxide (WO_3_) is a semiconductor and sensitive to CO_2_, it is a useful material for CO_2_ detection. Based on mixed valence phases in tungsten oxide and molybdenum nanostructured thin films made by RF reactive magnetron co-sputtering at 400 °C, Basyooni et al. [42] demonstrated a rapid responding CO_2_ sensor at room temperature. Three sample films were developed, namely, S1-WO_3_, S2-Mo_0.2_W_0.8_O_3_, and S3-Mo_0.4_W_0.6_O_3_. The films completed successful CO_2_ detection testing at room temperature (20 °C). When exposed to CO_2_, the S3-Mo_0.4_W_0.6_O_3_ sensor film exhibits Schottky contact with quick recovery time and reaction time upon activation by UV light. Lower concentrations, 2 and 0.5 sccm of CO_2_ at RT, were detected by the S3-Mo_0.4_W_0.6_O_3_ film in the dark and in UV light, respectively. The S3-Mo_0.4_W_0.6_O_3_ film exhibits a rapid recovery time and reaction time of 6.53 and 8.05 s for 0.5 sccm with a sensitivity of 29.19% when exposed to UV light (Figure 10f).

Thus, metal oxide semiconductors offer significant advantages like selectivity, high sensitivity, and stability, showing enormous potential for CO_2_ sensing. Additionally, the surface chemistries and band gap energies of various materials vary, which affects their sensing performance. Metal oxide-based CO_2_ sensors can precisely measure gas concentrations by utilizing their semiconductor qualities to modify electrical conductivity or optical characteristics, such as via doping with additional metals or creating heterostructures. To further improve selectivity, sensitivity, and response time, current research focuses on optimizing sensor design, fabrication, material synthesis, and operating conditions.

The most pristine MOS sensors, particularly SnO_2_-based ones, demonstrate poor selectivity and require high operating temperatures (typically 240–300 °C) for optimal performance. This presents significant limitations for practical applications, especially in portable and low-power devices. Another critical issue is the inconsistent response of MOS sensors under varying humidity conditions. This paper reveals that humidity can significantly impact sensor performance, either enhancing or diminishing the detection capabilities depending on the specific metal oxide material used. For instance, with CuO nanoparticles, the work function response decreases as humidity levels rise, introducing variability in sensing performance. Additionally, this paper highlights the challenge of achieving rapid response and recovery times, which are crucial for real-time monitoring applications. Unmodified MOS sensors often exhibit slow response and recovery times, limiting their utility in scenarios requiring immediate detection of CO_2_ concentration changes. To address these challenges, researchers have explored several innovative solutions. For example, La-doped SnO_2_ demonstrated significantly improved sensitivity (1.52) compared to undoped SnO_2_ (1.11). Similarly, Ni-doped SnO_2_ nanoparticles showed remarkably high sensitivity (62–73.29) with a fast response time of just 4 s for 100 ppm CO_2_. The doping strategy modifies the surface properties of the metal, facilitating improved gas adsorption and reaction kinetics. Creating heterojunction structures represents another successful solution pathway. This paper describes how combining materials with different electronic properties, such as SnO_2_-CdO and CaO-ZnO heterojunctions, facilitates enhanced charge separation and heightened response to CO_2_ gas. The SnO_2_-CdO heterostructure achieved a response of 10.29 to 1400 ppm CO_2_ at room temperature, which is 2.5 times higher than without illumination. Similarly, CaO-ZnO heterostructures showed a notable sensitivity of 26 at 150 °C for concentrations ranging from 100 ppm to 10,000 ppm. Morphological optimization has also proven effective in improving sensor performance. Researchers have synthesized various nanostructures like nanoflakes, nanowires, nanocomposites, nanopowders, and nanorods to increase the surface area for gas interaction. ZnO nanoflakes, for instance, demonstrated an exponential response with a sensitivity of 0.1135 for 600 ppm CO_2_ and rapid response and recovery times of 9–17 s in the 400–1025 ppm range. The application of external stimuli, particularly light activation, has been shown to enhance sensor performance significantly. The document mentions that UV light activation improved the detection capabilities of Mo_0.4_W_0.6_O_3_ films, achieving a rapid recovery time and reaction time of 6.53 and 8.05 s for 0.5 sccm with a sensitivity of 29.19%. Similarly, a light-induced SnO_2_-CdO sensor showed a 2.5 times higher response compared to the same sensor without illumination. Balancing temperature and humidity effects has been identified as a crucial approach for optimizing sensor performance. The document notes that increased temperature can counteract humidity-related performance variations in CuO nanoparticles, making the sensor’s response more consistent across different environmental conditions.

## 5. Recent Progress on Functionalization and Heterostructures

### 5.1. Noble Metal-Decorated Metal Oxide Semiconductor Sensors

**Figure 11:**
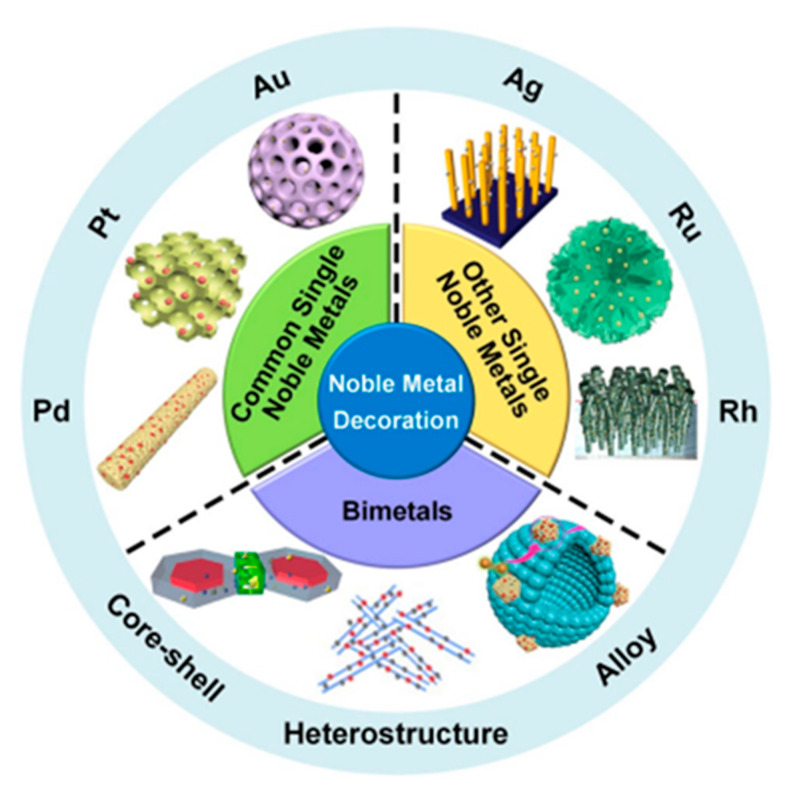
An overview of various noble metal-decorated metal oxide sensors for sensing [114].

Noble metal-decorated metal oxides are encouraging materials for CO_2_ sensing due to the combined effects of the materials, which enhance the catalytic properties of noble metals. A distinct class of metallic elements identified for their remarkable resistance to chemical corrosion and oxidation, with palladium (Pd), Platinum (Pt), and gold (Au) being the most popular due to their filled d-electron shells and prominent electrochemical nobility, are recognized as noble metals. Gold has the highest nobility and oxidation resistance, while platinum and palladium are pivotal for industrial applications due to their magnificent catalytic properties and high-temperature stability. Silver (Ag), though less noble and susceptible to tarnishing, still sustains exceptional resistance to corrosion in contrast with non-noble metals. Ruthenium (Ru) and rhodium (Rh), elements of the platinum group, are unusual but worthy for specialized catalytic applications despite being slightly more reactive than Pt, Pd, and Au. The differentiation between common and other noble metals is prominently based on their chemical inertness, abundance, and practical applications, as described in Figure 11. The catalytic properties of noble metals facilitate the conversion of CO_2_ into valuable compounds and enhance the adsorption of CO_2_. The sensing mechanism of noble metal-decorated metal oxides involves a complex interplay of surface interactions and catalytic activity, significantly improving sensitivity and selectivity. Moreover, the synergistic effect of integrating noble metals such as gold or platinum onto the surfaces of metal oxide sensors enhances overall sensing performance and gives a fast response, as shown in Table 4 [114].

The composition of sensors using metal oxide materials like tin oxide (SnO_2_), titanium dioxide (TiO_2_), and zinc oxide (ZnO) provides a stable substrate with a high surface area, enhancing the noble metals’ catalytic abilities. These metal oxides offer high particle surface area and chemical reactivity, facilitating the viable adsorption of gas particles. The interatomic properties of metal oxides with CO_2_ particles, through both chemical and physical interactions during detection, lead to changes in electrical conductivity or other measurable properties. Additionally, these metal oxides provide energy barriers for reactions and quicker responses to changes in CO_2_ concentration, with the synergistic effect improving the sensor’s sensitivity and response time. Furthermore, noble metals enable precise CO_2_ detection across diverse environments, enhancing sensor specificity and promoting CO_2_ adsorption over other gases.

### 5.2. Single Noble Metal-Decorated Metal Oxide Semiconductor Sensors

**Figure 12:**
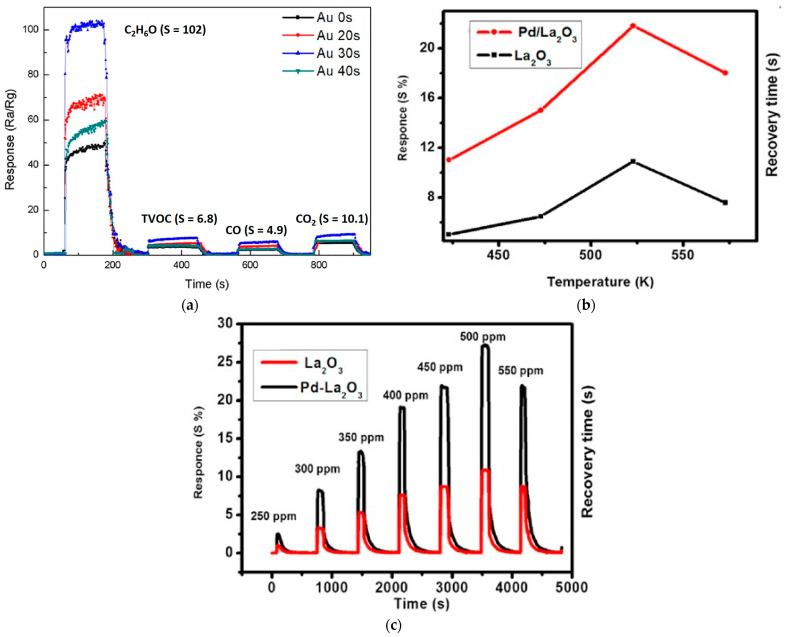
(**a**) Au on 5% La_2_O_3_/SnO_2_ NFs sputtered for 0, 20, 30, and 40 s at 300 °C to sense 100 ppm CO_2_ [115]. (**b**) Response of sensor to CO_2_ gas at various operation temperatures. (**c**) Response of Pd-La_2_O_3_ with La_2_O_3_ thin films with different concentrations [64].

A single noble metal-decorated metal oxide sensor represents a cutting-edge advancement in gas detection mechanisms. By integrating noble metals such as gold, platinum, or palladium onto the surface of metal oxide semiconductors, these sensors achieve enhanced sensitivity and selectivity in identifying carbon dioxide (CO_2_) gas. As technology advances, gas sensors are becoming smaller, more sensitive, and faster, while maintaining improved stability. Metal oxide materials like SnO_2_ and ZnO have been extensively investigated for gas sensors, with SnO_2_ being an early choice for sensor creation. To further improve sensor fabrication techniques, researchers have explored doping metal oxides with noble metals. Hsu et al. [115] studied nanofibers of SnO_2_ doped with 5 wt% La_2_O_3_ (La_2_O_3_/SnO_2_ NFs), synthesized through electrospinning and subsequently annealed at 600 °C for 1 h. The resulting gas sensor, utilizing these La_2_O_3_/SnO_2_ NFs, showed significant improvements in gas response, especially to CO_2_. Additionally, sputtering Au particles, measuring around 15–20 nm, onto the 5 wt% La_2_O_3_/SnO_2_ NFs further increased the gas detection capabilities, leading to a 50% improvement in sensor response, as shown in Figure 12a. The highest response value for CO_2_ was 10.1 s. Moreover, palladium (Pd) also demonstrates efficiency in CO_2_ gas sensing, contributing to enhanced detection and response. The impact of palladium decoration on CO_2_ gas detection capabilities was examined by Yadav et al. [64], who created thin films of La_2_O_3_ through spray pyrolysis techniques and decorated them with Pd nanoparticles using the successive ionic layer adsorption and reaction (SILAR) approach. CO_2_ gas detection capabilities of Pd-La_2_O_3_ and La_2_O_3_ were examined over various CO_2_ concentrations of 500 ppm and operating temperatures, as shown in Figure 12b. La_2_O_3_ exhibited a peak response of 13% at 523 K when exposed to CO_2_ gas, which increased from 13% to 28% following Pd enhancement (Figure 12c). Furthermore, the Pd-La_2_O_3_ film demonstrated increased selectivity to CO_2_.

### 5.3. P–N Heterojunction MOS

Carbon dioxide (CO_2_) is a chemically stable gas [117], making its detection more difficult using pristine metal oxides. However, the use of composite/combined MO semiconductors remarkably enhances sensor performance. When designing semiconducting composites, heterojunction interfaces are the most critical consideration, which are categorized into three types. N–n heterostructured materials are those that are created via bridging between electron-rich species, commonly known as n-type semiconductor substances, which alter the concentration of electrons and the mobility of carrier charge by an exchange of molecules among gases. Although metal oxide sensors have the potential for sensing gases, there is minimal difference in potential barriers and limited alterations during a transfer of charge in metal oxide sensors due to restricted selectivity and sensitivity. The notable obstacle deals with an existing identical electronic zone of the elements, which limits their potential to form exceptional alterations when revealed to various gaseous atmospheres in their electrical properties. p–p heterojunctions involve hole-based charge carrier species prominently comprising two p-type semiconductor substances. These heterostructures, when disclosed to molecules of gases, often struggle with the formation of important differences in the potential of interstitial molecules, which results in the least changes in electrical properties. These heterojunctions constrain their performance in applications of gas sensing due to major restriction, which arises from an analogous arrangement of energy bands and very nominal dynamics of electron–hole recombination. p–n heterostructured materials have the most premier gas sensing spatial arrangement, recognized by their powerful incorporated electric field generated by unsymmetrical structure stretching from p-type to n-type semiconductor materials. Not like n–n and p–p heterojunctions, which transfer only a small amount of charges, these junctions have the ability to improve the separation among charges and provide premium reactivity to gas molecules. The important disconnected arrangement of energy bands permits complicated reactions with particular gases; as an outcome, this provides enhanced susceptibility and identification abilities. Ultimately, these heteromaterials have the most advantageous arrangement and are extensively used for gas sensing technologies. Among these, the p–n junction is the most well-known interface used to modulate the properties of gas sensing [118]. Integrating a p–n heterojunction into MOS sensors enhances detection efficiency, allowing precise and rapid monitoring. In a p–n heterojunction, the flow of electron transfer goes from n-type to p-type, and holes move in the opposite direction until the system reaches the Fermi level of equilibrium. Since an n-type often has a higher Fermi level than a p-type, the formation of thick space charge layers related to the p–n junction narrows electrical transport channels and increases resistance [5]. When CO_2_ is added, electrical transport channels enlarge due to the acceptance of electrons by the oxidizing gas, which increases resistance and enhances gas sensing capabilities. This occurs because the initial high R_a_ and significantly increasing R_gas_ contribute to enhanced performance.

**Figure 13:**
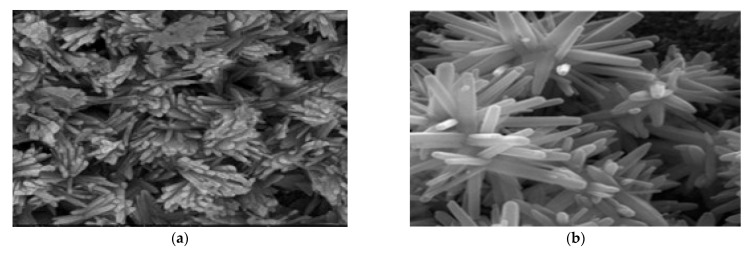

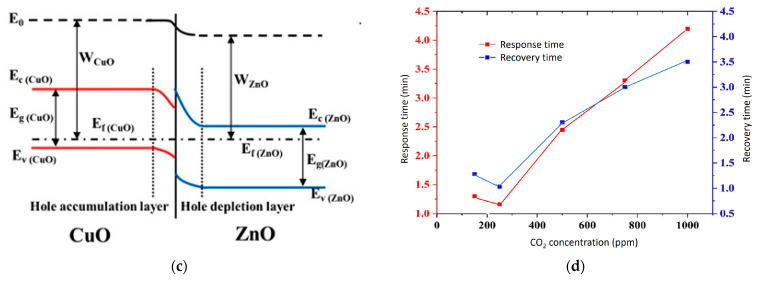
(**a**,**b**) The hierarchical ZnO/CuO nanorods image, hydrothermally grown. (**c**) Energy band of p–n heterojunction and ZnO-CuO heterojunction. (**d**) Recovery and response time for CO_2_ concentration [111].

The most commonly used p–n heterojunctions for CO_2_ sensing include MO-based semiconductors such as zinc oxide (ZnO), tin oxide (SnO_2_), and tungsten oxide (WO_3_), which are n-type materials, and copper oxide (CuO) and nickel oxide (NiO), which are p-type materials. Apart from this, it is known that SnO_2_ and ZnO are well-known semiconducting materials for sensing with complementary properties. Shravanti Joshi et al. [119] synthesized a CuO/SnO_2_ heterojunction by the hydrothermal route, incorporating 0.5% silver. The morphological analysis conducted using TEM not only furnished details about the dimensions and form of materials but also disclosed that the hierarchical structure of the CuO-SnO_2_ nanocomposite remained unaltered. It was investigated that the sensor shows superior sensitivity with short recovery and response times at 10,000 ppm concentration for CO_2_ gas in the air. It also showed the highest response of 72.02% at 300 °C for 1% CO_2_ gas, that is, 10,000 ppm in balanced air. The cross-sensitivity of the sensor (CuO-SnO_2_ 0.5 wt.% Ag) at low temperatures clearly shows that the sensor is more sensitive to CO_2_ compared to other gases. The morphology of the CuO-SnO_2_ heterostructure, like SnO_2_ and ZnO, is also promising. Keerthana et al. [111] prepared a ZnO-CuO heterojunction for sensing CO_2_ at RT using the hydrothermal method for synthesizing hierarchical ZnO/CuO nanorods. The surface morphology of ZnO was observed by SEM, which showed uniformly distributed spherical-shaped grains at 95 °C, and Figure 13a,b shows the nanorod structure of the grown ZnO-CuO. The work function of CuO, having a bandgap of 5.1 eV, is greater than ZnO’s 4.5 eV, resulting in the easy movement of electrons from higher to lower; because of this, the ZnO-CuO heterojunction gives good adsorption and desorption properties, as illustrated in Figure 13c. In further experiments, the heterojunction showed a better response for CO_2_ at an RT of 9.7% to 1000 ppm. Regarding recovery and response time, this sensor shows fast recovery and response times. Figure 13d shows that the recovery and response times for CO_2_ were 1.3 min, 1.16 min, 2.45 min, 3.3 min, and 4.2 min and 1.28 min, 1.03 min, 2.3 min, 3 min, and 3.5 min, respectively. These results have a great impact on room-temperature-based sensors, providing advanced sensing and better sensitivity to CO_2_ gas.

**Figure 14:**
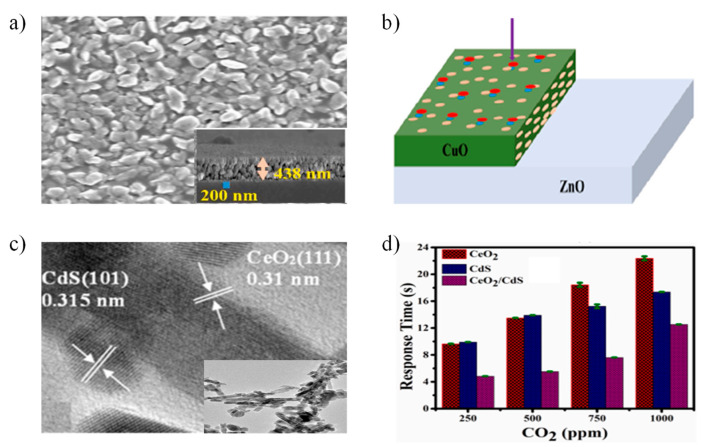
(**a**) Thin films of CuO-ZnO bilayer; (**b**) thin film of C/Z bilayer illustrate top CuO porous layer [121]; (**c**) TEM image of CeO_2_/CdS heterostructure [122]; (**d**) response time curves for CO_2_ at various concentrations [120].

Apart from SnO_2_- and ZnO-based heterojunction sensors, the newly studied CeO_2_/CdS heterojunction sensor shows great sensitivity to CO_2_. However, the morphology of CeO_2_/CdS illustrated in Figure 14a has a bilayer thin film with a porous top CuO layer, as shown in Figure 14b. Singh et al. [120] developed nanospheres of CeO_2_-CdS by a two-step hydrothermal technique, shown in Figure 14c. The sensor showed a response for various concentrations ranging between 250 and 1000 ppm. The higher response obtained at 1000 ppm is 3.62, which is almost 1.63 and 2.15 times greater than CdS and CeO_2_, respectively. Also, the recovery and response times are faster for CeO_2_-CdS than for pristine CdS and CeO_2_. Figure 14d shows that, for 250 ppm CO_2_, the recovery and response times are 4.82 s and 6.39 s, respectively.

Traditional metal oxide semiconductor sensors often struggle with poor response characteristics when exposed to CO_2_, necessitating innovative approaches to enhance their performance. To overcome these challenges, researchers have explored noble metal-decorated metal oxide semiconductor sensors as a promising solution. This section highlights how noble metals like gold (Au), platinum (Pt), palladium (Pd), silver (Ag), ruthenium (Ru), and rhodium (Rh) enhance the catalytic properties of metal oxide substrates, facilitating improved CO_2_ adsorption and conversion. For instance, Au-decorated La_2_O_3_/SnO_2_ nanofibers demonstrated a 50% improvement in sensor response, with the highest response value for CO_2_ at 10.1 s. Similarly, Pd-decorated La_2_O_3_ showed a significant enhancement in response from 13% to 28% at 523K when exposed to CO_2_ gas, along with increased selectivity. Another critical solution explored in this paper is the development of p–n heterojunction structures. These heterojunctions create powerful incorporated electric fields due to their asymmetrical structure stretching from p-type to n-type semiconductor materials. Unlike n–n and p–p heterojunctions, which transfer only small amounts of charges, p–n junctions significantly improve charge separation and provide superior reactivity to gas molecules. When CO_2_ is introduced to a p–n heterojunction sensor, electrical transport channels enlarge due to the acceptance of electrons by the oxidizing gas. This increases resistance and enhances gas sensing capabilities because of the initially high Ra and significantly increasing R_gas_. Specific examples of successful p–n heterojunctions include CuO/SnO_2_ (with 0.5% silver incorporation), which showed superior sensitivity with short recovery and response times at a 10,000 ppm concentration for CO_2_ gas, achieving the highest response of 72.02% at 300 °C. ZnO-CuO heterojunctions developed for room-temperature CO_2_ sensing demonstrated a response of 9.7% to 1000 ppm with fast recovery and response times of 2.3 min and 1.3 min, respectively. Additionally, newly studied CeO_2_/CdS heterojunction sensors showed promising results with a response of 3.62 at 1000 ppm, which is 1.63 and 2.15 times greater than pristine CdS and CeO_2_, respectively, with impressively fast recovery and response times of 4.82 s and 6.39 s for 250 ppm CO_2_.

## 6. Sensing Mechanism in Chemiresistive CO_2_ Sensors

**Figure 15:**
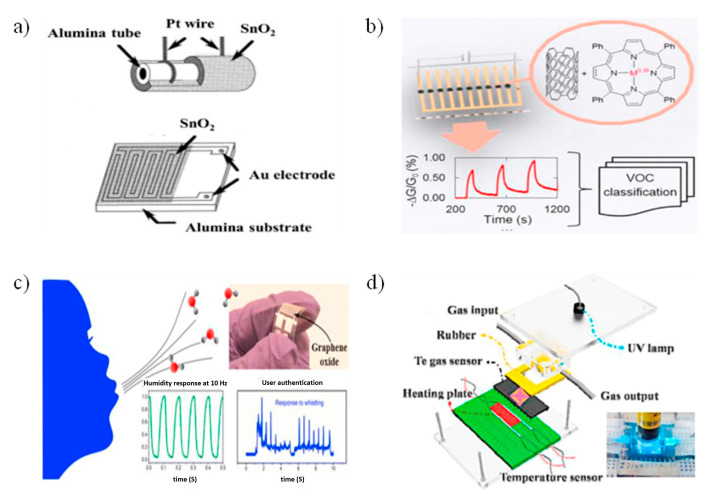
Representation of chemiresistive gas sensor devices: (**a**) Conventional prototype of gas sensors. (**b**) Chemiresistive sensor arrays employing porphyrin-functionalized single-walled carbon nanotubes. (**c**) Flexible sensor based on graphene oxide. (**d**) Chemiresistive sensor utilizing UV-assisted tellurium nanotubes [5].

Primarily, the manufacturing of chemiresistive devices involves depositing metal electrodes on substrates, which can be either planar or cylindrical, such as Al_2_O_3_ or silicon wafers [123], as shown in Figure 15a. Furthermore, the variety of chemiresistive sensors is expanding rapidly, including sensor arrays, flexible sensors, and UV-assisted sensor systems (Figure 15b, 15c, and 15d), respectively, which have emerged in recent years. The structures of these sensor devices complement traditional sensor technologies, offering a broad range of possibilities for the future advancement of sophisticated sensor devices. Understanding the sensing mechanisms of these diverse arrays is crucial for developing methods capable of generating gas sensing films with various structures and morphologies.

There is substantial interest in understanding the theories that explain sensing behaviors, yet opportunities and challenges remain in exploring the fundamental mechanisms underlying CO_2_ sensing. This context provides an overall overview of CO_2_ gas sensing mechanisms based on n-type semiconductors, where CO_2_ is considered an oxidizing gas. Mainly, there are two fundamental theories that explain the mechanisms: the oxygen vacancy model and the ionosorption model. All experimental observations are encompassed by both the ionosorption and oxygen vacancy models. These models serve as essential frameworks for comprehending the connections between nanomaterial structures and their sensing capabilities.

### 6.1. Ionosorption Model

**Figure 16:**
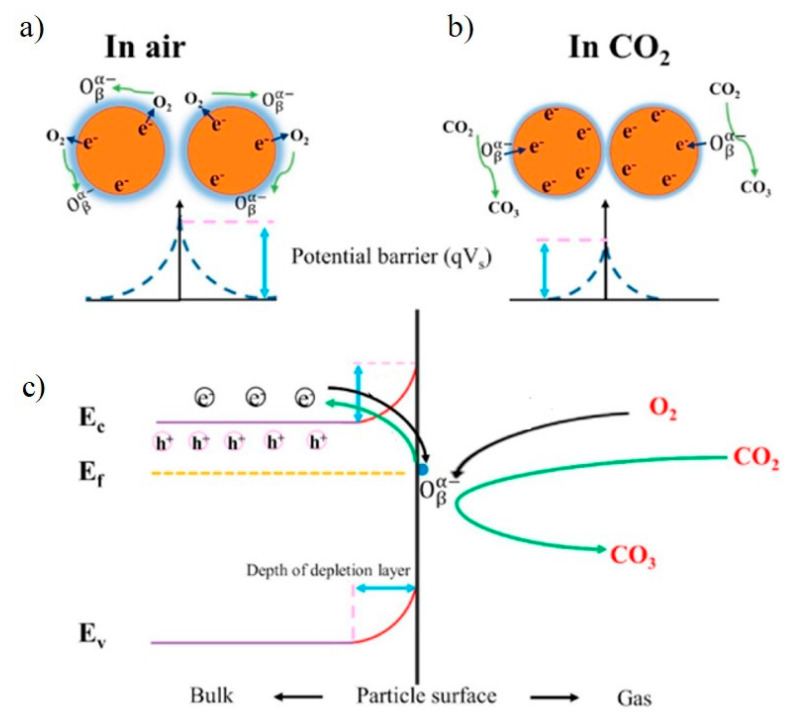
Illustration of the energy level and interaction reaction of n-type semiconductor; (**a**) depicts the state before exposure to CO_2_, while (**b**) represents the state after exposure to CO_2_. Additionally, (**c**) provides a schematic diagram of the corresponding band structure [5].

Unlike the oxygen vacancy model, the ionosorption model is more frequently utilized. In this model, the gas detection process is divided into reception and transduction sub-processes. Oxygen ions are formed primarily in the form of O^2−^ (below 420 K) or O^−^ (between 420 and 670 K) in ambient temperature. Above 670 K, the formation of concurrent O2- occurs, which is directly integrated into the lattice above 870 K. The electron delocalization leads to the collection of oxygen ions on the sensing layer’s surface, as illustrated in Figure 16, forming what is known as the depletion layer (Figure 16a) [124]. This induces band-bending, where the valence band (VB) and conduction band (CB) move upward relative to the Fermi level, generating a potential barrier on the surface (Figure 16b,c). Consequently, the convergence of two grains of two depletion layers produces the Schottky barrier, and the characteristics of this barrier are affected by the conductivity of materials, which can be changed by surface reactions [92,95,96].

Upon the introduction of CO_2_, molecules interact with pre-adsorbed oxygen ions on the surface of the semiconductor, leading to the formation of a meta-stable compound (CO_3_) with a brief existence. Electrons previously captured by oxygen are released onto the semiconductor, consequently raising the concentration of electrons in n-type semiconductors. Given that CO_2_ reacting with oxygen ions on the surface directly enhances the variability in conductivity, the changes in electrical parameters of CO_2_ sensors can be employed to detect the concentration of CO_2_ gas [125,126].

(5) $$\frac{\beta }{2}O_{2(g)}+{\alpha e}^{-}↔O_{\beta (ad)}^{\alpha -}$$

(6) $${CO}_{2(g)}+\frac{1}{\beta }O_{\beta (ad)}^{\alpha -}↔{CO}_{3(ad)}+\frac{\alpha }{\beta }e^{-}$$

The Debye length (δ) is commonly referred to as the depth of the electron depletion layer. The relationship between δ and grain size (D) effectively enhances the sensitivity of materials by optimizing the creation of depletion layers at grain boundaries and forming potential barriers between adjacent grains, which improves electron transport modulation at the time of gas exposure [97]. The impact of grain control is featured in the maximization of the surface-to-volume ratio, where smaller grains lead to an improved electron depletion effect and quicker gas molecule diffusion via grain boundaries, simultaneously increasing the sensor’s capability to measure CO_2_. The variation in the conductivity of the semiconductor is established by the thickness of the surface depletion layer. When grain size is significantly larger than twice the Debye length (2δ), sensitivity is relatively low because the EDL only contains a small portion of the sensing material, and variations in the potential barrier in the presence of CO_2_ do not significantly disrupt the overall conductivity of the sensing layer. Moderate sensitivity is anticipated since variation in conductivity is primarily enhanced by the width of the conduction channel, which is regulated by the concentration of oxygen ions on the surface of the sensing layer when D is greater than 2δ. When D ≤ 2δ, high sensitivity is achieved, as the depletion layer occupies the entire grain, and the concentration of oxygen ions affects the entire semiconductor [127]. Despite the widespread use of this model, it is important to note that there is limited spectroscopic evidence collected in situ to ascertain the contribution of oxygen ions during gas sensing [128].

### 6.2. Oxygen Vacancy Model

The study of the oxygen vacancy model has been extensively reviewed by Gurlo et al. [129]; a brief introduction to this model is provided here, using n-type SnO_2_ as an example. In SnO_2_, oxygen vacancies act as electron donors, and its conductivity is closely related to its non-stoichiometry (SnO_2−x_, 0 < x < 2). These oxygen vacancies (V-O) are introduced into the bulk of SnO_2_ due to non-stoichiometry [77]. In the absence of oxygen, the presence of these vacancies (V-O) significantly influences sensing activities. When CO_2_ is introduced, a cyclical process of reduction and reoxidation occurs on the SnO_2_ surface, leading to fluctuations in surface conductivity, which are crucial for sensing. Specifically, CO_2_ reacts with the oxygen vacancies (V-O), forming a neutral oxygen vacancy (Vx_0_). This neutral oxygen vacancy (Vx_0_) then ionizes, releasing electrons into the semiconductor and resulting in a decrease in sensor resistance. When oxygen is present, a significant portion of these oxygen vacancies (V-O) converts into lattice oxygen (Ox_0_). Upon the introduction of CO_2_, it reacts with the lattice oxygen (Ox_0_), creating a meta-stable compound (CO₃) and neutral oxygen vacancies (Vx_0_). These neutral oxygen vacancies then undergo ionization [102,103].

Numerous studies have been conducted to utilize gas sensing performance based on this theory; however, various issues still require resolution. One such challenge is the role of vacancy diffusion in the bulk of the metal oxide, which is highly dependent on the material and temperature, necessitating further investigation. Additionally, the mechanisms of surface reoxidation and reduction in metal oxide-based gas sensors within the operational temperature range of 250 to 450 °C are not fully understood. Specifically, the surface reoxidation and reduction kinetics of a SnO_2_-based gas sensor at 250 to 450 °C are relatively sluggish compared to its observed low response times [101,105]. This finding suggests that other processes, such as chemisorption, may also play a significant role in gas sensing activities, enhancing the device’s overall ability.

## 7. In-Depth Examination

### 7.1. Challenges

Performance. The development of a chemiresistive sensor for carbon dioxide (CO_2_) sensing, particularly using nanomaterials, faces significant challenges that shape future research directions. Achieving optimal sensitivity and selectivity is difficult due to interference from various gases and environmental conditions. Ensuring long-term stability is crucial, as many nanomaterials may degrade over time, affecting reliability. Real-world environments, with their temperature and humidity variations, complicate sensor performance, necessitating robust sensor architectures and compensation mechanisms. Manufacturing. Additionally, the high cost and scalability of manufacturing processes for nanomaterial-based sensors remain significant considerations, especially for large-scale industrial applications. Environmental Safety. Environmental and ethical concerns are paramount. The use of nanomaterials raises issues regarding their potential release into the environment and their impact on human health and ecosystems. Responsible sensor development must address these concerns to avoid unintended consequences. Technical issues. Standardization and reproducibility present ongoing challenges, as consistent sensor performance across different batches is essential for industrial applications. Navigating the regulatory landscape is another significant challenge, requiring collaboration between researchers, industry stakeholders, and regulatory bodies to adapt existing regulations for nanomaterial-based sensor technologies.

### 7.2. Future Prospective

Technology Integration. Looking ahead, integrating Artificial Intelligence (AI) and Machine Learning (ML) into sensor systems is a key direction for enhancing CO_2_ sensing capabilities. This integration can improve response times, data interpretation accuracy, and adaptive learning for better performance in dynamic environments. Sustainability Focus. Sustainability is a crucial focus, with trends directed toward using eco-friendly nanomaterials and green technology. Researchers aim to develop materials from renewable sources with minimal ecological footprints, contributing to environmentally conscious manufacturing methods. Enhancing selectivity for multi-gas detection is another important trajectory. Technical Advancements. Researchers are focused on improving chemiresistive sensors to simultaneously sense multiple gases, which is useful in various industries including industrial safety and environmental monitoring. Energy efficiency is also a key focus, with efforts to design sensors that reduce energy consumption, improve battery life, and incorporate energy harvesting mechanisms, particularly for autonomous and remote sensing applications. Future advancements may involve integrating chemiresistive sensors with carbon-based nanomaterials like carbon nanotubes, graphene, and nanodiamonds, which offer unique electronic properties and large surface areas, enhancing sensitivity and selectivity. Hybrid nanomaterials, combined with functional materials like metal oxides or polymers, show synergistic effects that improve sensing performance. Advanced fabrication methods, such as 3D printing, offer cost-effective and scalable production. Application Development. Integrating CO_2_ sensors into wearable devices and IoT (Internet of Things) applications accelerates trends toward continuous, real-time monitoring. Advancements in miniaturization, portability, and connectivity with smart devices are necessary for user-friendly and accessible CO_2_ sensors. Industry Development. Standardization and regulatory compliance will play crucial roles in future sensor development, ensuring consistency, quality, and reliability in CO_2_ monitoring. Cross-disciplinary collaborations between engineers, data scientists, and environmental scientists will drive innovative solutions, addressing challenges and pushing the boundaries of CO_2_ sensing capabilities.

## 8. Conclusions

This comprehensive review of chemiresistive sensors for CO_2_ detection has highlighted the importance of understanding their principles, definitions, operational mechanisms, key parameters, and advantages. Our examination has emphasized the pivotal role played by nanomaterials in CO_2_ sensing, showcasing the rich possibilities for tailoring sensor performance through various categories of nanomaterials, including metal oxides, carbon-based nanomaterials, nanocomposites, and hybrid nanomaterials. Innovative functionalization strategies, sensitivity enhancement methodologies, advancements in miniaturization, integration, and diverse applications have all contributed to recent progress in CO_2_ sensor technology. However, it is necessary to acknowledge the limitations and challenges associated with CO_2_ sensors. Issues such as stability, selectivity, and reproducibility remain, necessitating the development of more sophisticated CO_2_ sensors. Regarding the real-world availability of CO_2_ sensor technology, NDIR is an excellent technology, which serves as a prominent application of commercialized CO_2_ sensors that provides accurate data from indoor air quality monitoring to industrial process control. Increasing environmental insights and severe rules and regulations on the quality of air among numerous fields, such as agriculture, healthcare, building management, automobiles, electronics, etc., are forcing the rapid growth of CO_2_ sensors in the global market to reach USD 1.2 billion by 2027 with a 7.5% compound annual growth rate. Applications extending from enhancing air flow management and regulating environmental conditions to filtering and cleaning exhaust systems and scientific cultivation strategies are consequences of the sensors that are rapidly compressed and synchronized with networked intelligence. Revolutionary technologies are quickly enhancing sensors’ potential by lowering manufacturing expenses, optimizing wireless infrastructure, and minimizing structural complexity, which increases their capability among multiple commercial enterprises. As global awareness about environmental safety and air quality continues to increase, CO_2_ sensors are becoming essential elements in medical surveillance, ecological solutions, etc.

While previous reviews have laid important groundwork for understanding metal oxide-based CO_2_ sensing, this paper reveals several promising research directions that demand immediate attention. Future development of CO_2_ gas sensing technology should focus on addressing three critical challenges: achieving reliable room-temperature operation, enhancing long-term stability in varied environmental conditions, and improving selectivity in complex gas mixtures. It proposes that heterostructure engineering presents the most promising path forward, particularly through precise control of interface phenomena between p-type and n-type materials where electron depletion layers can be strategically manipulated to amplify CO_2_ sensitivity. It indicates that p–n heterojunctions involving CuO/SnO_2_ and ZnO/CuO show exceptional promise, with potential for further optimization by controlling the interfacial area and crystallographic orientation at the junction. Our analysis suggests that combining hierarchical nanostructures with selective noble metal decorations (especially Au and Pd at concentrations below 1 wt%) could create synergistic effects that dramatically lower operational temperatures while maintaining fast response times. It shows that Pd-decorated La_2_O_3_ nanoparticles demonstrate a 115% improvement in sensitivity compared to undecorated counterparts, suggesting a mechanistic pathway involving the catalytic activation of CO_2_ molecules. Further exploration of bimetallic decorations (such as Au-Pd or Pt-Ru combinations) could potentially overcome the current limitations in response times, which remain above 1 s for most reported systems. Beyond materials optimization, we identify integration with emerging technologies as a crucial frontier. Specifically, the combination of metal oxide sensors with flexible substrates could revolutionize wearable environmental monitoring, while integration with AI-driven signal processing could enable intelligent compensation for cross-sensitivity and drift issues that have historically limited practical applications. Preliminary calculations suggest that implementing deep learning algorithms for pattern recognition in sensor response could improve selectivity by up to 85% in multi-gas environments without requiring additional hardware modifications. The miniaturization trend in sensor technology presents another promising direction, with MEMS-based platforms potentially reducing power consumption by two orders of magnitude compared to conventional heating elements. Our analysis of recent advancements in microfabrication techniques indicates that interdigitated electrode designs coupled with localized heating can achieve operating temperatures of 300 °C while consuming less than 10 mW of power. Additionally, we observe that in situ characterization techniques capable of monitoring surface reactions during CO_2_ sensing remain underdeveloped, presenting a significant opportunity for fundamental mechanistic insights that could guide rational sensor design. Based on our comprehensive review of 145 diverse sources spanning various advanced materials, we contend that future research should prioritize multifunctional sensing platforms that combine the catalytic properties of noble metals with the electronic benefits of well-defined heterostructures. The development of core–shell nanostructures, where a catalytic metal oxide shell encapsulates a highly conductive core, represents a particularly promising approach that merits further investigation. Such architectures could potentially achieve sub-second response times while operating at near-ambient temperatures.

In the future, the development trajectory of CO_2_ sensors is poised for significant advancements. Breakthroughs in the preparation of novel nanomaterials and the refinement of synthesis techniques, along with the integration of advanced signal processing techniques, will play pivotal roles in minimizing the limitations of current sensors. The integration of sensors into various industrial and environmental applications underscores the transformative potential of these methods. Looking forward, pushing the boundaries of materials, processing signals, interdisciplinary collaboration, and application domains will shape the future landscape of CO_2_ sensors. Continuous development, innovation, collaborative efforts, problem-solving strategies, and modifications stand to revolutionize our approach to environmental monitoring and CO_2_ detection, making substantial contributions to the realization of a more sustainable and bright future.

## Figures and Tables

**Table 2:** The comparative study between CO_2_ and other oxidizing and reducing gases [89,90].

Gas Type	Typical Sensor Response	Temperature Sensitivity	Typical Sensing Mechanism	Selectivity	Sensing Efficiency
CO_2_	Weak interaction	Low	Minimal surface adsorption	Poor	Limited
Reducing Gases (H_2_, CO)	Strong response	High	Surface catalytic reactions	Good	High
Oxidizing Gases (O_2_, NO_2_)	Strong response	High	Surface charge transfer	Good	High

**Table 3:** Illustrating key properties and applications of n-type and p-type MOS sensors.

Metal Oxide	Type	Key Properties	Applications
SnO_2_	n-type	High sensitivity, excellent chemical stability, low cost.	Indoor air quality monitoring, environmental sensing
TiO_2_	n-type	High-temperature stability, reversible changes in resistance.	Outdoor air quality monitoring, automotive sensors
ZnO	n-type	High surface reactivity, good conductivity.	Environmental monitoring, industrial leak detection
In_2_O_3_	n-type	Good sensitivity, stability.	Optimized for specific gas interactions.
Fe_2_O_3_	n-type	Cost-effective, abundant.	Industrial and agricultural environments
CuO	p-type	Independence from relative humidity.	Low-cost portable applications, automotive sensors
NiO	p-type	Lower response compared to n-type oxides but potential for improvement through doping.	Automotive exhaust, air quality sensors

**Table 4:** Summary of CO_2_ gas sensors based on noble metal-decorated MO gas sensor.

MO Sensor	T (°C)	Response/Recovery (s)	Concentration (ppm)	Reference
Pd-La_2_O_3_-nanoparticles	250	0.83/-	500	[64]
Au/La_2_O_3_/SnO_2_-nanofibers	300	10.1/-	100	[115]
La_2_O_3_-SnO_2_-film	225	0.32/-	1000	[116]
La_2_O_3_-SnO_2_-film	225	4.38/-	1000	[116]
Au La_2_O_3_-SnO_2_-nanofibers	300	10/-	100	[115]

## Data Availability

This paper includes the unique findings discussed in the research. For further details, please reach out to the corresponding author.
